# COVID-19 and the Heart: A Systematic Review of Cardiac Autopsies

**DOI:** 10.3389/fcvm.2020.626975

**Published:** 2021-01-28

**Authors:** Ashraf Roshdy, Shroque Zaher, Hossam Fayed, John Gerry Coghlan

**Affiliations:** ^1^Critical Care Unit, Whipps Cross University Hospital, Barts Health NHS Trust, London, United Kingdom; ^2^Critical Care Department, Faculty of Medicine, Alexandria University, Alexandria, Egypt; ^3^Department of Pathology, Mohammed Bin Rashid University of Medicine and Health Sciences, Dubai, United Arab Emirates; ^4^National Pulmonary Hypertension Unit-Cardiology Department, Royal Free Hospital, London, United Kingdom; ^5^Institute of Cardiovascular Science, UCL, London, United Kingdom

**Keywords:** COVID-19, SARS-CoV-2, post-mortem, cardiac injury, autopsy

## Abstract

**Importance:** Severe acute respiratory syndrome coronavirus 2 (SARS-CoV-2)-associated cardiac injury has been postulated secondary to several mechanisms. While tissue diagnosis is limited during the acute illness, postmortem studies can help boost our understanding and guide management.

**Objective:** To report the cardiac tissue autopsy findings in coronavirus disease 2019 (COVID-19) decedents.

**Evidence Review:** Articles published in PubMed and Embase reporting postmortem cardiac pathology of COVID-19 decedents till September 2020. We included adult studies excluding preprints. The Joanna Briggs Institute Critical Appraisal Checklist for Case Reports was used to assess quality. We extracted gross and histology data as well as the incidence of myocarditis, cardiac ischemia, thrombosis, and dilatation. We also looked at the reported cause of death (PROSPERO registration CRD42020190898).

**Findings:** Forty-one relevant studies identified including 316 cases. The deceased were mostly male (62%) and elderly (median age, 75; range, 22–97 years). The most common comorbidities were hypertension (48%) and coronary artery disease (33%). Cardiac pathologies contributed to the death of 15 cases. Besides chronic cardiac pathologies, postmortem examination demonstrated cardiac dilatation (20%), acute ischemia (8%), intracardiac thrombi (2.5%), pericardial effusion (2.5%), and myocarditis (1.5%). SARS-CoV-2 was detected within the myocardium of 47% of studied hearts.

**Conclusions and Relevance:** SARS-CoV-2 can invade the heart, but a minority of cases were found to have myocarditis. Cardiac dilatation, ischemia, mural, and microthrombi were the most frequent findings. The systematic review was limited by the small number of cases and the quality of the studies, and there is a need to standardize the cardiac postmortem protocols.

## Key Points

- **Question**: What are the pathological cardiac findings in postmortem autopsies of COVID-19 patients?
- **Findings**: The systematic review included 41 studies and 316 cases. Apart from chronic pathological findings, postmortem examination demonstrated cardiac dilatation (20%), acute ischemia (8%), intracardiac thrombi (2.5%), pericardial effusion (2.5%), and myocarditis (1.5%). SARS-CoV-2 was detected within the myocardium of 47% of studied hearts.
- **Meaning**: The main pathological findings in patients dying during the acute COVID-19 illness were cardiac dilatation, ischemia, and (micro)thrombosis. Myocarditis was a rare finding in this cohort of patients.

## Introduction

While coronavirus disease 2019 (COVID-19) primarily affects the lungs, it is increasingly recognized as a multiorgan disease. The underlying mechanism may be direct viral invasion or secondary to the systematic effect of the infection (e.g., hypoperfusion, hypoxia, massive inflammatory response/cytokine storm).

Cardiac comorbidity and standard coronary risk factors (e.g., obesity, diabetes, and hypertension) are associated with adverse outcomes among patients with COVID-19 (1). COVID-19 is also associated with release of the highly specific marker of myocardial cell death—Troponin. Where this is tested in all hospitalized patients, the prevalence of elevated Troponin has been reported in up to 71% and is a predictor of outcome (40% mortality vs. 8% in those without myocardial injury) (2). A recent meta-analysis of published retrospective observational studies identified a positive troponin in 27% of 1,550 patients, with a similar impact on increased mortality and increased probability of needing intensive care (3).

Acute setting cardiac imaging (mainly echocardiography), while a valuable tool to assess the cardiac function and structure, suffers many limitations (4). Endomyocardial biopsies (EMBs) are rarely performed due to logistics and infection control reasons.

Postmortem examination (PM) is a valuable resource to understand the pathophysiology, cause of death, and the extent of organ involvement. Lessons from previous infectious diseases [e.g., human immunodeficiency virus (HIV)] have demonstrated the benefit of PMs (5).

To date, single case reports to modest-sized autopsy series have failed to clarify the nature of cardiac involvement. Histological findings vary from interstitial edema with or without myocarditis (6), lymphocytic endothelialitis (7), microvascular microthrombi and venous thrombosis (8), to extensive interstitial fibrosis with no endothelialitis (9), and no evidence of myocarditis (10). Optimal management depends on knowledge of the mechanism of myocardial injury, as the treatment and required follow-up will differ among the various pathologies outlined above.

To gain a better understanding of the prevalent cardiac findings in patients dying of COVID-19—we undertook a systematic review of all reported autopsies that included cardiac findings.

## Methodology

A protocol of a systematic review was registered on PROSPERO database (CRD42020190898) on the 23rd June 2020. The aim was to investigate autopsy findings for patients who died from a confirmed COVID-19 infection (https://www.crd.york.ac.uk/prospero/display_record.php?RecordID=190898).

**Figure 1:**
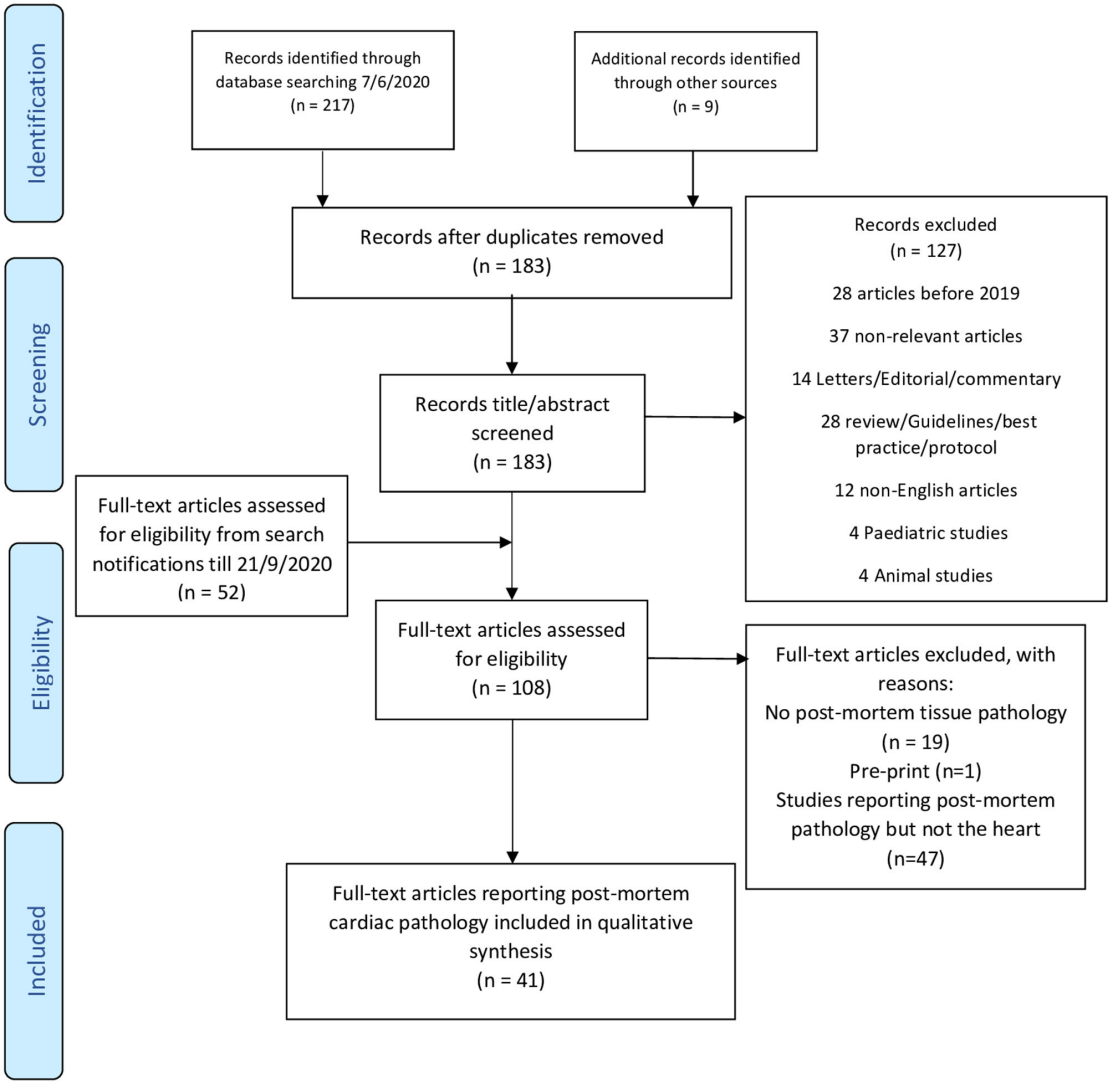
PRISMA 2009 flow diagram. Adapted from (11).

An initial systematic search was conducted through the *NHS Healthcare Databases Advanced Search* tool (HDAS) on 7th of June 2020 for published articles in PubMed and Embase databases. The search strategy is shown in Table 1. An electronic search alert was set to identify any new study on the EMBASE database through Healthcare Databases Advanced Search (HDAS) (option not available for PubMed) till the 21st of September 2020. The search was done by AR and included the period from 1st January 2019 to the search date. AR screened the references for additional articles. We identified 88 articles that reported PM tissue pathology. AR reviewed the full-text to retrieve articles which reported PM cardiac pathology. We reviewed only published articles in journals (excluding pre-prints) in the English language and included humans since 2019 (Figure 1: PRISMA diagram). Articles or cases with duplicate reporting have been excluded to the best of our knowledge. AR assessed the quality of the case series studies using the Joanna Briggs Institute Critical Appraisal Checklist for Case Reports (12) (Supplementary Table 2). SZ and AR extracted the data from the included studies. Any conflict was resolved by discussion and mutual agreement.

**Table 1:** Search strategy.

**Search**	**Query**
**PubMed**
#1	(COVID*).ti,ab
#2	(SARS-CoV-2).ti,ab
#3	(Coronavirus 2019).ti,ab
#4	(nCOV 19).ti,ab
#5	(1 OR 2 OR 3 OR 4)
#6	(autopsy).ti,ab
#7	(necropsy).ti,ab
#8	(post-mort*).ti,ab
#9	(postmort*).ti,ab
#10	(histolog*).ti,ab
#11	(6 OR 7 OR 8 OR 9 OR 10)
#12	(5 AND 11)
**Embase**
#13	(COVID*).ti,ab
#14	(SARS-CoV-2).ti,ab
#15	(Coronavirus 2019).ti,ab
#16	(nCOV 19).ti,ab
#17	(13 OR 14 OR 15 OR 16)
#18	(autopsy).ti,ab
#19	(necropsy).ti,ab
#20	(post-mort*).ti,ab
#22	(postmort*).ti,ab
#23	(histolog*).ti,ab
#24	(18 OR 19 OR 20 OR 21 OR 22)
#25	(17 AND 23) [DT 2019–2020] [English language] [Human age groups Adult 18–64 years OR Aged 65+ years] [Humans]

### Patient, Intervention, Comparison, and Outcome (PICO) Statement

#### Patient

Adult patients (≥18 years old) who died and had a laboratory confirmation of severe acute respiratory syndrome coronavirus 2 (SARS*-*CoV*-*2) infection.

#### Intervention

None.

#### Comparison

None or other patients who died from another cause.

#### Outcome

Pathological description of PM cardiac involvement.

## Results

### Search Strategy

**Figure 2:**
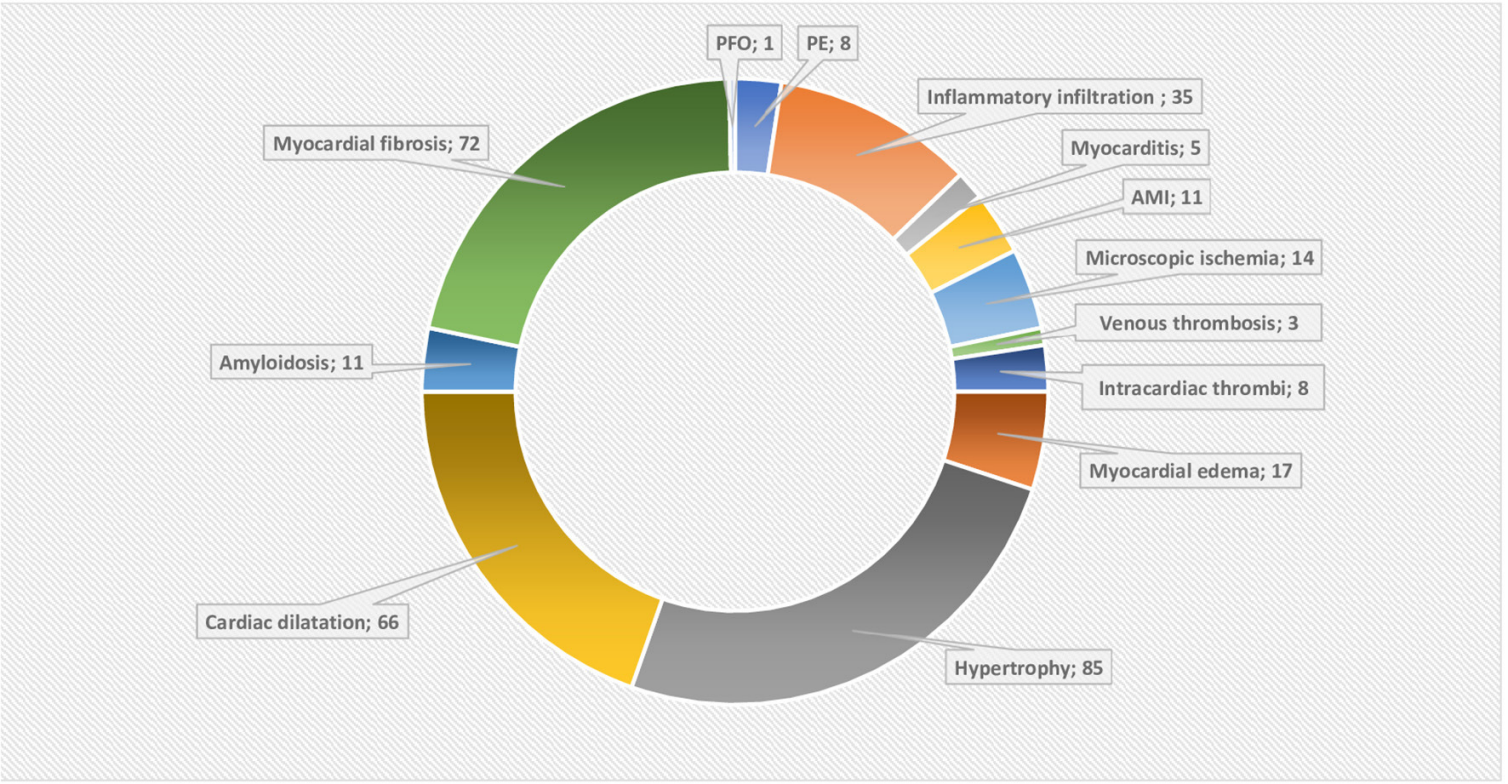
Doughnut chart showing the reported post-mortem acute and chronic pathologies. AMI, Acute myocardial infarction; PE, pericardial effusion; PFO, patent foramen ovale. The data labels show the number of reported acute or chronic pathologies, note that they can overlap in the single patient. Chronic pathologies include hypertrophy and amyloidosis, while myocardial fibrosis, pericardial effusion and dilatation can be acute or chronic. The rest are considered as acute pathologies.

The search resulted in 226 titles. After duplicate removal of and title screening, we screened the full text of 108 articles (52 from weekly alerts) that yielded 88 articles reporting PM tissue pathology. Among those, 41 studies reported PM heart examination and included 336 cases (Figure 1). Studies were mostly case reports (*n* = 13) or case series (*n* = 24), while three studies compared cases to controls (6–10, 13–48) (Table 2, Figure 2). Authors reported cases from 14 countries, mostly developed westernized ones (Supplementary Table 1). Two studies reported on the same population, with one mainly focusing on PM cardiac examination (22, 23). The quality of the included studies was mostly moderate (Supplementary Table 2).

We analyzed the PM cardiac histopathology for 316 cases [after excluding cases unconfirmed as COVID-19 (*n* = 6) or with no PM cardiac tissue examination (*n* = 14)].

### General Characteristics of the Studies

Study characteristics and pathological findings are detailed in Supplementary Tables 1, 2, respectively. Cases were predominantly male (172/275, 62%). The deceased were mostly elderly [median: 75 years; interquartile range (IQR), 63–84 years; range, 22–97 years, *n* = 228] and overweight [body mass index (BMI): median, 27; IQR, 22.9–34.7 kg/m^2^; range = 15.4–61.2 kg/m^2^, *n* = 148).

### Comorbidities

Cardiovascular comorbidities were prevalent, most commonly hypertension (*n* = 152, 48.1%), coronary artery disease (CAD) (*n* = 105, 33.2%), cardiomyopathy and heart failure (*n* = 68, 21.5%), and atrial fibrillation (AF) (*n* = 35, 11.1%). Other comorbidities included chronic respiratory diseases (*n* = 91, 28.7%), diabetes mellitus (*n* = 81, 25.6%), chronic kidney disease (CKD) (*n* = 53, 16.7%), dementia (*n* = 40, 12.7%), and cancer (*n* = 39, 12.3%).

### Timing

The median duration of prehospital symptoms (*n* = 82) and hospital stay (*n* = 158) were 5 (IQR, 2–7) and 6 days (IQR, 3–10), respectively. In total, the median duration from the onset of symptoms to death was 12 days (range, 0–52 days, *n* = 98). The median time interval between death to PM autopsy was 1.2 days (*n* = 31).

### Pathological Findings

Cardiac abnormalities either on gross pathology or histology were identified in almost all cases. Most autopsies demonstrated chronic cardiac pathologies [hypertrophy (*n* = 85), fibrosis (*n* = 72), and amyloidosis (*n* = 11)], which may have contributed to the increased heart weight where this was reported (median, 455 g; IQR, 399–576 g; range, 250–1,070 g, exceeded normal range in 39/44 (normal reference: male, 270–360 g; female, 200–280 g)] (47).

**Table 2:** Postmortem pathology findings in the included studies.

**References**	**Number of cases**	**Autopsy technique**	**Time from death to autopsy**	**Postmortem pathology**					**Cause of death**
				**Gross pathology/heart weight**	**Histology and microscopy**	**Tissue SARS-CoV-2**	**Myocarditis (*n*)**	**Acute ischemia (*n*)**	
Duarte-Neto et al. (6)^*^	10	Ultrasound-guided minimally invasive autopsy	N/A	N/A	**Related to comorbidities: (*****n*****)** • Cardiomyocytes hypertrophy (9) • Myocardial fibrosis (9) • Previous MI (4) **Acute:** • Mild lymphomononuclear myocarditis (2) • Fibrin microthrombi (2) • Interstitial edema (9)	N/A	2	0	N/A
Schaller et al. (13)	10	Autopsy	N/A	N/A	4/10 mild lymphocytic myocarditis (no true myocarditis) 2/10 epicarditis	N/A	0	0	N/A
Buja et al. (14)	3	Autopsy	N/A	**P1**: • Weight 420 g • CA: patent with minimal atherosclerosis • LV wall thickness: 1.1 cm • RV wall thickness: 0.2–0.3 cm **P2**: • Weight: 1,070 g • 4-chamber hypertrophy and dilatation • CA: patent with minimal atherosclerosis • LV wall thickness: 1.5–1.6 cm • RV wall thickness: 0.5 cm **P3**: • Weight: 670 g. • CA: minimal atherosclerosis, widely patent • Both ventricles were dilated • Thickness of LV free wall and IVS was 1.6 cm and that of the RV was 0.3 cm	**P1**: Microscopy: • Cardiomyocytes with moderately enlarged hyperchromatic nuclei • Individual cardiomyocytes with vacuolar degenerative change • No evidence of inflammatory infiltrate indicative of myocarditis **P2**: **Histology:** • Epicardial lymphocytic infiltrates • Cardiomyocyte hypertrophy • Multifocal interstitial and replacement fibrosis • Scattered damaged individual cardiomyocytes • No inflammatory foci indicative of myocarditis **P3**: • Multifocal lymphocytic infiltrates in epicardium • CMC -enlarged hyperchromatic nuclei • Individual CMC—changes of acute injury • No inflammatory cellular infiltrates found • Prominent foci of CMC disarray—superior portion of the IVS • Intramural coronary arteries—intimal and medical thickening with luminal narrowing • Both diagnostic features of hypertrophic cardiomyopathy • Random sections—sinoatrial and atrioventricular conduction system—no abnormalities	N/A	0	0	N/A
Yan et al. (15)	1	Autopsy	18 h after death	Heart weight: 410 g**Gross:** • Streaking of right atrial wall myocardial tissue: thin myocardial trabecula alternating with areas of epicardium lacking underlying myocardial tissue • No CAD	• LV: No focal lesions suggestive of acute or chronic hypoxic injury • RV: dilated • Mild myxoid edema • Mild myocyte hypertrophy • Rare foci of lymphocytes in myocardium No evidence of viral myocarditis	N/A	0	0	N/A
Lax et al. (10)	11	Autopsy	N/A	**P1**: Myocardial hypertrophy, myocardial fibrosis, endocardial thrombi LV **P2**: Myocardial hypertrophy, coronary small vessel disease myocardial fibrosis **P3**: Myocardial hypertrophy, coronary small vessel disease myocardial fibrosis, thrombosis of a myocardial vein **P4**: Myocardial hypertrophy, coronary small vessel disease myocardial fibrosis **P5**: Myocardial hypertrophy, myocardial fibrosis **P6**: Myocardial hypertrophy, myocardial fibrosis **P7**: Myocardial hypertrophy, coronary small vessel disease myocardial fibrosis **P8**: Myocardial hypertrophy, coronary small vessel disease myocardial fibrosis **P9**: Myocardial hypertrophy **P10**: Myocardial hypertrophy, coronary small vessel disease myocardial fibrosis, amyloidosis **P11**: Myocardial hypertrophy, myocardial fibrosis, Focal lymphocytic infiltrate • Myocardial hypertrophy 11/11 • Coronary small vessel disease 6/11 • Myocardial fibrosis 10/11 • No viral myocarditis In 10 patients, both ventricles were massively dilated In 1 patient, intraventricular endocardial mural thrombi without ischemic changes of adjacent myocardium No acute myocardial necrosis or inflammatory changes found except 1 patient with focus of fragmented cardiomyocytes with lymphocytic and granulocytic reaction		N/A	0	0 (1 venous thrombus with no ischemia)	N/A
Lacy et al. (16)	1	Autopsy with minor modifications	N/A	• Weight: 438 g • Moderate coronary atherosclerosis in each of the main coronary distributions, no occlusions or critical stenoses • Myocardium: no obvious infarct, firm texture, and red-brown color. • LV thickness: 1.2–1.4 cm • Cardiac valves: normal	• Myocyte hypertrophy • No acute ischemic changes • Interstitial and perivascular fibrous tissue • No viral myocarditis • Moderate infrarenal aortic atherosclerosis	N/A	0	0	**Autopsy:** ARDS due to viral pneumonia due to COVID-19
Wichmann et al. (17)	12	Complete autopsy	P1: 1 day P2: 1 day P3: 2 days P4: 1 day P5: 2 days P6: 1 day P7: 4 days P8: 1 day P9: 4 day P10: 5 days P11: 2 days P12: 3 days	Mean heart weight: 503 g (median, 513 g) **P1:** 660 g, eccentric hypertrophy of both ventricles **P2**: 515 g, CAD with stenting, post-MI, cardiac aneurysm **P3:** 510 g, biventricular hypertrophy, moderate CAD **P4**: 605 g, LVH **P5**: 360 g, CAD, post-MI **P6**: 250 g, normal **P7**: 415 g, CAD, moderate hypertrophy, mitral ring calcification, post MI, pacemaker, lipomatous cordis **P8**: 575 g, CAD, post bypass surgery, post-MI cardiac aneurysm, global hypertrophy **P9**: 355 g, left atrial dilatation, CAD, post-MI **P10:** 390 g, CAD, post-MI **P11:** 650 g, CAD, post aortic valve replacement, biventricular hypertrophy **P12:** 745 g, CAD, hypertrophy	Lymphocytic myocarditis: 1/12	In 5 of the patients, viral RNA detected in other tissues (heart, liver, or kidney) in concentrations exceeding viremia	1	0	N/A
Menter et al. (18)	21	Full body autopsy in 17 cases Partial autopsy in some (?) *in-corpore technique*	Mean PMI from death to autopsy: 33.3 h (11–84.5 h)	• Hypertrophy: 15/21 • Senile cardiac amyloidosis: 6/21 • Peracute myocyte cell necrosis: 3/21 (sequelae of shock) • Acute MI−1/21		N/A	0	1 (acute MI) 3 peracute myocyte cell necrosis	N/A
Varga et al. (7)	3 (1 excluded as still alive)	Autopsy	N/A		•No lymphocytic myocarditis • Endotheliitis **P1**: • Inflammatory cells associated with endothelium and apoptotic bodies **P2**: • Lymphocytic endotheliitis • Acute posterior myocardial infarction • No viral lymphocytic myocarditis	N/A	0	1 (acute posterior MI)	N/A
Tian et al. (19)	4 (2 heart biopsies)	Needle core biopsies of lung, liver, and heart	N/A		Heart biopsies obtained from P 1 and 4 **Both:** • Focal mild edema • Interstitial fibrosis • Myocardial hypertrophy • No inflammatory cellular infiltration • Endocardia and myocardia—no inflammatory cellular infiltration • Focally, myocardium irregular in shape with darkened cytoplasm—not sufficient for acute myocardial injury • Focal interstitial fibrosis, and myocardial hypertrophy	RT-PCR assay for SARS-COV-2: Positive for P1 and negative for P4	0	0	N/A
Barton et al. (20)	2	Autopsy	N/A	**P1**: • Heart weight: 402 g • No adhesions, effusions, or thrombi • CAD: marked 2 vessels **P2**: • Heart weight: 372 g • No adhesions, effusions, or thrombi • CAD: mild • Aorta intimal fatty streaking	**P1**: Microscopic: acute ischemic injury Abdominal aorta atherosclerosis no evidence of myocarditis **P2**: No myocarditis	N/A	0	1 (microscopic acute injury)	**Autopsy:** **P1**: COVID-19, with CAD listed under “other contributing factors.” **P2**: complications of hepatic cirrhosis,” with muscular dystrophy, aspiration pneumonia, and COVID-19 listed as other significant conditions
Conde et al. (21)	1	Autopsy	N/A	• Mild stenosis of aortic valve • Slight increase LV thickness • Dilatation of both ventricles		N/A	0	0	Severe bilateral CAP
Edler et al. and Lindner et al. (22, 23)	80 (74 pre-mortem and 6 post-mortem)	Full autopsy	**Days: n** 0d: 3 1 day: 9 2 days: 19 3 days:14 4 days: 12 5 days: 7 6 days: 1 7 days: 1 8 days: 3 9 days: 3 12 days: 2 15 days: 1 41 days: 1 n/a: 4	**P39:** MI + cardiac tamponade in 1 case *(despite COVID positive, authors noted death not related to COVID)*	**P4**: A small lymphocytic infiltrate in RV as a sign of myocarditis Chronic diseases changes—scarring in the myocardium	SARS-CoV-2 RNA in the myocardium: 24/39 • Viral load: >1,000 copies per μg RNA: 16/24 • <1,000 copies per μg RNA: 8/24 • Virus replication: 5/16 (among those with high viral load of SARS-CoV-2) (sub-analysis in subsequent study)^**^	1 (RV)	1	See Supplementary Table 1
Sekulic et al. (24)	2	Autopsy (P1 autopsy sine brain and spinal cord) (P2 chest and abdomen only per family request)	**P1**: autopsy 29 h after death **P2**: 39 h after death	**P1**: • Heart enlarged • Weight: 620 g • Chronic IHD: severe stenosis native CA (left anterior descending, left circumflex, and right main CA), patent graft vessels • Moderately extensive replacement-type interstitial fibrosis**P2**: • Heart enlarged • Weight: 560 g • LV hypertrophy, • Mild calcified atherosclerotic CAD	• **P1**: no significant findings • **P2**: none described	Lower levels of SARS-CoV-2 RNA detected in the heart of P1	0	0	**P1**: RF due to SARS-CoV-2 **P2**: SARS-CoV-2 infection leading to respiratory and multiorgan system failure
Suess et al. (25)	1	Autopsy	N/A	Accumulation of serous fluids in pericardial cavity (30 ml)	• Patchy non-specific pericardial infiltration including lymphocytes and plasma cells • No neutrophils/granulomas seen • No inflammatory infiltrate/substantial damage in the myocardium	N/A	0	0	ARDS due to severe DAD as a result of severe infection with SARS CoV-2.
Aguiar et al. (26)	1	Autopsy	N/A	• Heart weight: normal for BMI (460 g) • LV and IVS wall thickness: 1.3 cm • RV wall thickness: 0.3 cm • Fatty streaks: anterior interventricular branch of left CA	No signs of cardiac hypertrophy	N/A	0	0	**Pathology:** Pulmonary changes related to SARS-CoV-2 and high fever without secondary bacterial infection
Fox et al. (27)	10 (African American)	Autopsy (cardiac examination in 9 cases)	N/A	**P2**: 420 g **P3**: 550 g **P4**: 540 g **P5**: 480 g **P6**: 370 g **P7**: 420 g **P8**: 450 g **P9**: 340 g **P10**:600 g • Myocardium: firm, red-brown, and free of significant lesions in all patients • Mild to moderate serosanguinous pericardial and pleural effusions (*n =* ?) • CA: no significant stenosis or acute thrombus formation • Most significant was cardiomegaly and RV dilatation. In several patients, massive dilatation could be seen; for example, in one case, RV cavity was 3.6 cm in diameter and the LV was 3.4 cm at its greatest diameter	**Microscopic examination:** • Myocardium: no large/confluent areas of myocyte necrosis • Scattered individual myocyte necrosis • In rare areas, lymphocytes adjacent to, but not surrounding, degenerating myocytes • May be early manifestation of viral myocarditis, but no significant brisk lymphocytic inflammatory infiltrate suggestive of viral myocarditis	LM: No viral cytopathic effect, but direct viral myocardial infection cannot be ruled out by this limited examination	0	0	COVID-19 (Withdrawal of care)
Beigmohammadi et al. (28)	7 (5 with cardiac tissues)	Core needle biopsies	n/a		**P1**: • Few scattered lymphocytes and mastocytes without evidence of myocyte necrosis or degeneration • No myocarditis **P3**: • All inflammatory cells positive for CD68; but none stained with CD3 • No myocarditis • No evidence of myocyte necrosis • Ischemic process of cardiac muscle highly suggested **P5**: • Severe interstitial infiltration of LCA-positive inflammatory cells with predominance of CD68 positive macrophages and focal aggregation of CD3 positive T cells • Histologic evidence of myocyte necrosis including hyper-eosinophilia and enucleation • Ischemic necrosis of myocardium should be considered **P6**: • No interstitial inflammation **P7**: • Majority of inflammatory cells showed immunoreactivity for CD68 and rare cells positive for CD3. • No myocarditis • No evidence of myocyte necrosis • item Ischemic process of cardiac muscle highly suggested	N/A	0	3 (suggested)	N/A
Wang et al. (29)	2	Autopsy	**P1**: 6 h **P2**: 9 h	No obvious gross abnormalities	• Multifocal myocardial degeneration and myocardial atrophy and interstitial fibrous tissue hyperplasia Few scattered CD20-positive B cells and CD3-positive T cells	No obvious viral infection in parenchymal cells using IHC with antibodies against Rp3-NP.	0	0	Respiratory and circulatory failure in both
Rapkiewicz et al. (8)	7 vs. 9 controls died from ARDS from other cause	Autopsy + Tissue+ IHC + EM	N/A		In all cases, megakaryocytes associated with fibrin microthrombi within the cardiac microvasculature Venous thrombosis in 2 hearts of P3 and P7 **P4**: • Focal inflammatory infiltrate composed of lymphocytes, mixture of Band T cells as per CD20 and CD3, with CD4 in greater number than CD8 • Associated myocardial necrosis in epi-myocardial region • Localized infiltrate • Diffuse, transmural pallor of the LV. Platelet microthrombi in the region of inflammation identified using CD 61 • No granulomas • Staining for complement (C4d) negative in all tested cases **P7**: • Intramyocardial venous thrombosis with septal MI despite only minimal coronary atherosclerosis • Elevated levels of antiphospholipid IgM Ab detected postmortem	No viral inclusions on EM of the heart in any of 4 cases analyzed (P 2, 4, 6, and 7)	0	MI 1/7 venous thrombi 2/7 (both high Troponin but only 1 with septal MI on gross examination) Early ischemic changes 3/7 Mural fibrin thrombi 2/7	N/A
Bösmüller et al. (30)	4	AutopsyTissue for virology and EM (4 cases)	Autopsy after 48 h for patient 1 and within 24 h for P 2, 3, and 4	**P1**: • Increased weight: 520 g • Biventricular dilatation • Coronary arteries: no sclerosis or signs of ischemia • Hyperplastic myocardium **P2**: weight 527 g **P3**: weight 411 g **P4**: weight 590 g		Significant levels of SARS-CoV-2 RNA in the lungs of all patients by qRT-PCR, but not in the hearts	0	0	**Clinical** P1: Pneumonia (Pathology: acute cardiac failure was considered the likely cause of death.) P2: ARDS, liver failure, shock P3: ARDS, liver failure, shock P4: ARDS, multiorgan failure
Schweitzer et al. (31)	1 (and 1 control)	Autopsy	N/A	• Weight: 340 g • CA: atherosclerosis with pre-existing narrowing to 50% of the lumen of both the left anterior descending and right coronary arteries • No macroscopic signs of myocardial ischemia	No relevant histological findings (such as contraction band necroses, infarction, or inflammation) noted	N/A	0	0	N/A ? severe ARDS
Xu et al. (32)	1	PM biopsy samples	N/A		No obvious histological changes seen in heart tissue	N/A	0	0	N/A
Youd et al. (33)	3	Autopsy	**P1**: 5 days **P2**: 8 days **P3**: 10 days	**P1**: • Minimal CA atheroma **P2**: • Enlarged heart • Weight: 592 g • CA: minimal atheroma **P3**: • Enlarged heart • Weight: 582 g • CA: focal significant stenosis by atheroma • Old myocardial scarring	No myocarditis	N/A	0	0	N/A
Bradley et al. (9)	14	Standard autopsy for 7 cases *In situ* dissection for 7 cases (3 cases: fresh tissue collection)	**n/a**	No endotheliitis and scarce microthrombi (focal pulmonary microthrombi were identified in five patients) **P1**: Interstitial fibrosis, myocyte hypertrophy **P2**: Interstitial fibrosis, myocyte hypertrophy, replacement fibrosis **P3**: Interstitial fibrosis, myocyte hypertrophy **P4**: Interstitial fibrosis, myocyte hypertrophy, replacement fibrosis **P5**: Interstitial fibrosis, myocyte hypertrophy **P6**: Interstitial fibrosis, myocyte hypertrophy **P7**: Interstitial fibrosis, myocyte hypertrophy, vascular predominant amyloid **P8**: Interstitial fibrosis, myocyte hypertrophy, replacement fibrosis Myocarditis (aggregates of lymphocytes surrounding necrotic myocyte. SARS-CoV-2 S protein immunohistochemistry was negative) **P9**: Interstitial fibrosis, myocyte hypertrophy **P10**: Interstitial fibrosis, myocyte hypertrophy, replacement fibrosis, subsegmental pulmonary embolus **P11**: Interstitial fibrosis **P12**: Interstitial fibrosis, myocyte hypertrophy, replacement fibrosis, subsegmental pulmonary emboli **P13**: Interstitial fibrosis, myocyte hypertrophy, replacement fibrosis, myocardial amyloid **P14**: Interstitial fibrosis, myocyte hypertrophy, replacement fibrosis		Viral RNA detected in the liver, heart, and blood for P8 and P13	1	0	See Supplementary Table 1
Ducloyer et al. (34)	1	Autopsy PMCT IHC	48 h	• Heart weight: 470 g • Moderate RV dilatation • No increase in myocardial wall thickness • Nonobstructive atherosclerotic plaques in CAs and aortic bifurcation	• Mild coronary artery atherosclerosis • No myocarditis • Scattered wavy fibers	Not done	0	0	
Cirstea et al. (35)	1	Autopsy IHC	N/A	Cardiomegaly with dilation of the RV and blood clots in the heart	• Recent intracardiac thrombosis • Vascular leukostasis with thrombi formation mainly in the small subepicardium vessels • Massive interstitial edema (obliterated the intercalated disks in between the myocardial cells) • Occasional scant mononuclear inflammatory cells and petechial hemorrhages	N/A	0	0	
Nicolai et al. (36)	1 (5 cases and 5 controls but only 1 with heart tissue)	Autopsy IHC	N/A	N/A	Inflammatory microthrombi. Neutrophil extracellular trap-like structures in heart specimens associated with fibrin deposition (1/1 patient)	N/A	0	0	
Grosse et al. (37)	14	Autopsy	N/A	• Myocardial hypertrophy (heart weight range, 385–750 g): 13/14	• Acute MI in 3/14 • Focal myocardial fibrosis 3/14 • Previous MI in 6 (42.9%) • Cardiac amyloidosis in 1 • Mild to severe CA atherosclerosis in 14/14: ➣ 2: mild 1-vessel coronary artery disease with 25% lumen stenosis, ➣ 6: 2-vessel coronary artery disease (25% lumen stenosis: *n =* 1; 25–50% lumen stenosis: *n =* 4; >75% lumen stenosis: *n =* 1), ➣ 6: moderate to severe 3-vessel coronary artery disease (25–50% lumen stenosis: *n =* 1; 50% lumen stenosis: *n =* 1; >75% lumen stenosis: *n =* 4 • All patients: some mononuclear inflammatory cells in myocardial interstitium, mainly CD3-positive T-lymphocytes (ranging in density from 2 to 4 lymphocytes/HPF)	N/A	0	3	
Schwenson et al. (38)	1	Autopsy	4 days	Heart enlarged weight: 380 g. RV: normal thickness (3 mm) LV: concentrically hyperplastic (23 mm)	Tissue samples normal No evidence of microthrombosis	N/A	0	0	
Remmelink et al. (39)	17	Autopsy	<5 days	• Cardiomegaly: 14/17 • Pericardial effusion: 2/17 • Atheromatosis: 8/17 (2- severe)	• Chronic ischemic cardiomyopathy: 15/17 • Acute MI: 2/17 • No evidence of contraction bands or myocarditis • Cardiac fibrosis: 5/17 • Chronic pericarditis: 1/17 • Abdominal aortic aneurysm: 1/17	Viral RNA detected by RT-PCR in heart tissue of 14/17	0	2	
Okudela et al. (40)	1	Autopsy	13 h	N/A	No remarkable changes	N/A	0	0	
Adachi et al. (41)	1	Autopsy	5 h	Heart weight: 420 g RV dilatation, with 10 ml of cardiac effusion	No notable changes	Not detected in heart	0	0	
Nadakarni et al. (42)	26 (focus on thromboembolism)	Autopsy	N/A	N/A	Microthrombi in heart: 4/26	N/A	0	0	
Dalahmah et al. (43)	1	Autopsy	3 h	N/A	The heart showed LVH, focal subendocardial fibrosis, but no myocarditis or ischemia	N/A	0	0	
Oprinca and Muja (44)	3	**P2**: full autopsy **P1 and P3**: thoraco-abdomino-pelvic autopsies	**P1**: 24 h **P2**: N/A **P3:** N/A	**P1**: Weight: 355 g Dilated cardiomyopathy, LVH, RA, and RV dilatation. Coronary atherosclerosis but preserved luminal permeability. Aortic atherosclerosis **P2**: Weight: 342 g RA and RV dilatation No morphological abnormalities of the myocardium, CA, or aorta **P3**: Weight: 412 g Ischemic cardiomyopathy LVH RA and RV dilatation. Severe coronary atherosclerosis. Aorto-coronary bypass. Complicated atherosclerosis	**P1:** • Mild to moderate perivascular edema • Vascular congestion • Areas of small contraction band-like lesions • Small number of scattered lymphocytes between the myocardial fibers **P2**: • Small vessel thrombosis • Marked vascular congestion • Mild edema between the muscle fibers • Myocardial fibers tend to form contraction bands **P3**: • Myocardosclerosis • Myocardial fibrosis due to old MI • Mild edema • Marked vascular congestion • Acute circulatory disorders ***Overall (P1–3)*** Small areas of contraction bands and scattered lymphocytes No signs of myocarditis *P2, P3: Pulmonary emdotheliitis (mild vasculitic reaction: lymphocytic invasion of pulmonary vascular wall with no fibrinoid necrosis)*	No microscopic signs of viral infection of myocardium	0	0	
Wang et al. (45)	1	Percutaneous biopsies (heart tissue in 1 patient among 3)	N/A		• Old MI • Hypertrophic myocytes • Fatty infiltration • Nuclear pyknosis • Interstitial edema and fibrosis • No viral myocarditis	N/A	0	0	
Jensen et al. (46)	2	Autopsy	9 days	**P1**: Foramen ovale fully closed. Aorta and its branches: mild atheroma **P2**: Foramen ovale was probe patent		N/A	N/A	0	
Elsoukkaryet al. (47)	30	Autopsy	5–382 h (median: 43)	Normal weight 2/30: Mean 350 g Cardiomegaly 28/30: Mean 490 g Heart Intramyocardial small vessel thrombi: 6/30 Valve-associated thrombi 2/30 Thrombosis and co-existing infarction: 1/30	• Atherosclerosis (>50% stenosis): 17/30 • Myocyte hypertrophy: 24/30 • Myocyte ischemia: 5/30 (1 with acute MI due to thrombosis into atherosclerotic plaque) • Interstitial fibrosis: 20/30	N/A	0	5 (1 acute MI)	
Hanley et al. (48)	10	9 full autopsies + 1 limited biopsy	Median: 6 days	• Median weight was high (450 g; IQR, 315–535 g) • LVH: 4/9 • RA thrombus: 1 • Pericardial effusion: 3 • Pericarditis: 2 (1 acute pericarditis + P5 showed florid fibrinous pericarditis containing fungal hyphae) • **P5**: Non-bacterial thrombotic (marantic) endocarditis (no known history or autopsy findings consistent with malignancy or chronic disorder associated with non-bacterial thrombotic (marantic) endocarditis). Disseminated mucormycosis and numerous other thrombotic features • **P8**: Cardiac amyloidosis and RA thrombosis • Macroscopic acute coronary thrombosis in right CA: 1/9	• Fibrinous pericarditis • with fungal hyphae • Non-bacterial thrombotic endocarditis • Thrombi in the microcirculation of the heart: 5/9 • CAD: negligible, 3/9; mild, 4/9; moderate, 2/9 • Acute myocardial ischemic damage (<24 h) noted in patient with acute coronary thrombus • **P2**: mottled myocardium and subendocardial contraction band necrosis; uncertain whether the contraction band necrosis was related to ischemia or inotropic medication in the ICU	PCR of viral E gene: 3/5 (P1,P2,P4)Sub-genomic viral RNA transcripts: 2/5 (P1, P2)	0	1 (± 1 with band necrosis of unknown etiology)	

* *Among 10 cases, one COVID-19 diagnosis based on radiological and pathological findings*.

** *A subanalysis of cardiac tissue histopathology had been subsequently published (37)*.

*Ab, antibodies; ARDS, acute respiratory distress syndrome; BMI, body mass index; CA, coronary artery; CAD, coronary artery disease; CAP, community-acquired pneumonia; CMC, cardiomyocytes; DAD, diffuse alveolar damage; EM, electron microscopy; ICU, intensive care unit; IHC, immunohistochemistry; IVS, interventricular septum; LM, light microscopy; LV, left ventricle; LVEF, left ventricular ejection fraction; LVH, left ventricular hypertrophy; MI, myocardial infarction; MR, mitral regurgitation; P, patient; PAD, peripheral arterial disease, PE, pulmonary embolism; PM, postmortem; PMI, postmortem interval; RA, right atrium; RF, respiratory failure; RV, right ventricle*.

While myocardial fibrosis was identified in only 72 cases, in a series where this was specifically reported, the prevalence was high (9, 10). Myocyte and ventricular wall hypertrophy were reported in 85 cases, again highly prevalent where specifically reported (18). Significant cardiac dilatation/cardiomegaly was described in 66 cases (10, 14, 15, 21, 24, 27, 30).

Overall changes consistent with cardiac ischemia and thrombosis were the most frequently reported acute findings. Acute myocardial ischemia was evident in 25 cases either in the form of acute myocardial infarction (MI) (*n* = 11) or microscopic evidence of acute or early ischemia (*n* = 14). Moreover, fibrin microvascular thrombi were identifiable in 27 cases (6, 8, 35, 36, 42, 47, 48). Thrombi in cardiac veins were described in three cases (8, 10). Lastly, there was eight cases with mural thrombi including the heart valves (*n* = 3) and the right atrium (RA) (*n* = 1) (10, 35, 47, 48).

### Viral Invasion of Myocardium

Twelve studies explored the presence of SARS-CoV-2 within the myocardium using different techniques (Table 2) (8, 9, 17, 19, 23, 24, 29, 30, 39, 41, 44, 48). In those studies, SARS-CoV-2 was detected in 50 of 105 hearts (47%). However, clear myocarditis meeting the Dallas criteria was described in only five cases (6, 9, 17, 22). In an additional 35 cases, minimal lymphocytic (*n* = 33) or mononuclear infiltration (*n* = 2) not meeting the criteria for myocarditis was identified (13, 15, 27, 28). In three cases, authors attributed those changes as consistent with ischemic damage response (28). Overall, lymphocytic infiltration was scarce but can be detected in any of the pericardium, myocardium, epicardium, or endothelium. Lastly, pericardial affection was described in the form of pericardial effusion (*n* = 8) and pericarditis (*n* = 5, one had chronic pericarditis).

### Cause of Death

The cause of death was reported for 190 cases and, for the majority of these, was respiratory in origin (Supplementary Tables 1, 2). However, cardiac contribution to death was mentioned for 15 cases while pulmonary embolism (PE) was mentioned in eight cases.

## Discussion

Our review confirms that among patients dying from COVID-19, cardiac abnormalities are prevalent, but that specific changes of acute myocarditis are uncommon (1.5% of cases). Myocardial ischemia, thrombosis, and cardiac dilatation were the most dominant acute findings (Figure 2). Prevalence of the non-specific myocardial edema (ME) was 100% in the six studies reporting it (6, 15, 19, 35, 44, 45). The highly prevalent chronic cardiac pathologies not only reflect the impact of cardiac comorbidities but also complicated the histopathological interpretation.

### Role of Ischemia, Endotheliitis, and Hypercoagulability

The most alarming finding is the intracardiac, coronary arterial, and venous thrombosis, which may be in part explained by the COVID-19-associated coagulopathy (CAC). Myocardial ischemia can be further aggravated by the frequent pre-existing CAD and myocardial supply–demand mismatch.

By means of its receptor, SARS-CoV-2 can directly invade the endothelium leading to endothelial cell (EC) inflammation (i.e., endothelialitis), dysfunction, and death (49). Endothelial dysfunction can also result from an inappropriate immune and cytokine response. Endothelialitis, and hence EC dysfunction, subsequently induces a procoagulant state (CAC), loss of barrier function, inflammatory tissue infiltration, edema, and injury (49, 50). Cardiovascular comorbidities are usually associated with chronic EC dysfunction, which can explain the worse outcome when further acute insult is superadded.

However, endothelialitis was not a consistent finding in our reviewed studies but, when detected, was associated with microthrombi and had multiorgan distribution. Varga et al. showed multiorgan endotheliitis in all three studied cases (7). Ackermann et al. showed widespread endotheliitis and capillary thrombosis in COVID-19-affected lungs in a much more common prevalence than in non-COVID acute respiratory distress syndrome (ARDS) lungs (51). In contrast, Bradley et al. concluded not only no evidence of endothelialitis but also little evidence of cardiac microthrombi (9). Rapkiewics et al. noted no endothelial abnormalities but a platelet-rich microthrombi in all seven hearts examined, despite anticoagulation (8). It appears that alternate mechanisms of ischemia overlap, and while anticoagulation may be highly relevant in limiting pulmonary thrombosis, this may be less likely to significantly ameliorate any cardiac contribution to poor outcomes. Nicolai et al. highlighted thrombi to be rich in platelets, fibrin, and neutrophil extracellular traps (NETs), while Jensen et al. described platelet-rich cerebral microangiopathy (36, 46). The role of NET and platelets may be significant and could support other potential therapies (e.g., antiplatelet therapy).

### Chamber Dilatation and Myocardial Edema

Heart weight exceeded the normal range in 90% of cases reflecting a combination of chronic pathologies (e.g., hypertrophy), myocardial edema (marker of injury), and chambers dilatation. The observed cardiac dilatation (especially of the right heart) may be long standing or acute and hence relate to preload or afterload (pulmonary hypertension) changes occurring during the acute illness and its treatment.

ME reflects myocardial tissue response to most types of injury and hence its nonspecificity. Ischemia, septic cardiomyopathy, viral, or inflammatory infiltration can all contribute to it. Schmittinger et al. showed ME in 90% of PM septic hearts in a patchy distribution (median of 25% of tissue sections) (52). Of note, ME can reflect an early tissue change after insult (as early as 3 min in the setting of ischemia due to the disruption of the Na+/K+ pump) (53). Detecting ME has therapeutic implications, as it causes less energetic efficiency, arrhythmias, and reduced cardiac wall compliance. All of these are expected to impair systolic and diastolic function and can ultimately lead to fibrosis (53, 54). While cardiac MRI (CMR) can detect it *in vivo*, histological diagnosis remains technically challenging (53). This challenge, combined with the lack of standardized protocol guidelines for PM cardiac pathology reporting, may mean that ME was overlooked in many of the published reports.

### Myocardial Fibrosis

Myocardial fibrosis was reported in nearly a quarter of cases. It is the end result of cardiac injury arising from different acute or chronic mechanisms. Cytokines were also implicated in cardiac fibroblast activation (55, 56).

The interpretation in COVID-19 is difficult and depends on many factors. It can reflect a chronic or a *de novo* subacute process. Aging and many reported comorbidities are strongly associated with fibrosis (56). Of note, amyloidosis (a pathology associated with fibrosis) was described in 11 cases and was significantly more prevalent when compared to a historical age-matched cohort (18, 37, 48).

Myocardial fibrosis can be divided into two types: interstitial fibrosis and replacement fibrosis, with considerable overlap between the two (55). While interstitial fibrosis is considered reactive and potentially reversible, replacement fibrosis is not (55). Interstitial fibrosis was previously detected in 100% of PM septic hearts but in a patchy nature (52). Such focal nature means that an extensive PM cardiac pathological examination is necessary. In fact, CMR may be superior as a diagnostic modality despite the difficultly to perform in unstable patients (55, 57).

Myocardial fibrosis represents the structural equivalent of heart failure. While ME is expected in the “reversible” septic cardiomyopathy, increased fibrous deposition (i.e., replacement fibrosis) would not be a likely finding in such reversible pathology (52, 57, 58).

### Viral Invasion, Inflammatory Infiltrate, and Myocarditis

Studies investigating the presence of SARS-CoV-2 within the myocardium were positive in about half the cases. In 1986, The Dallas criteria were proposed for the histopathological categorization and diagnosis of myocarditis based on endomyocardial biopsies. The “Dallas criteria” defines acute myocarditis as “an inflammatory infiltrate associated with myocyte necrosis or damage not characteristic of myocardial ischemia.” Borderline myocarditis requires a less intense inflammatory infiltrate with no light microscopic signs of myocyte destruction (59). In COVID-19 PM studies, inflammatory infiltrate (mainly lymphocytic) was observed in a minor proportion (about 10%) and was limited in extent for the majority of cases. As such, when interstitial edema and inflammatory infiltrate were observed, they did not meet the diagnostic criteria of myocarditis, except in five cases. In fact, some authors attributed such inflammatory infiltrate to an ischemic process (28). This suggests that contrary to early conjectures, acute and fulminant myocarditis are rare during the acute illness.

### Clinical and Imaging Correlation

Correlating the histopathological data to the clinical, imaging, and investigational data can provide more insights into the likely mechanisms of cardiac involvement in COVID-19. Clinical presentation varies from ST elevation MI due to thrombotic occlusion of epicardial coronaries, to ischemia and/or infarction without obstructive coronary disease, through to tachy and brady arrhythmias, depressed left and right ventricular function, and occasional pericardial involvement (60). A review of published literature suggests that elevated Troponin and heart failure dominate the clinical presentations (61).

Echocardiography is readily performed in the acute setting but provides limited insights into the cause when compared to CMR. In a large multinational survey, Dweck et al. reported the echocardiographic findings in 1,216 studies performed over 17 days (62). Fifty-five percent of scans were abnormal. Impaired LV function or dilation (39%) followed by RV abnormalities (33%) dominated. These findings are non-specific, but clear wall motion abnormalities suggesting infarction were rare (3%). The RV abnormalities most likely relate to increased afterload given the high prevalence of pulmonary thromboembolism and extensive lung damage associated with COVID-19 infection (63). The LV abnormalities are non-specific but provide further evidence of the high prevalence of cardiac damage.

CMR-based studies have focused on patients post recovery (too late for confirmation of myocarditis) but have shown a high prevalence of abnormalities. The largest to date is a German study of relatively young patients (mean age, 49 years), largely managed at home (67 of 100), studied a median of 71 days post infection. Seventy-eight percent were reported to show abnormalities, including reduction in LV function, elevated T1 and T2 (the latter suggesting ME) and late gadolinium enhancement (LGE) (non-ischemic pattern in 20, ischemic in 12). Three patients with very elevated T2 were referred for endomyocardial biopsy and typical features of myocarditis reported. The T1 and T2 abnormalities suggest ongoing myocardial edema, and the LGE enhancement suggests fibrosis—both of which are common in the autopsy data (64).

The second CMR-based study included only patients in whom Troponin had been elevated during hospital admission. Fifty-one patients were studied 27 days post hospital discharge. In 22 patients, pulmonary embolism and/or coronary ischemia were identified before scanning as the most likely cause of troponin leakage. Among 29 patients (mean age, 64 years) with no clinically identified cause for myocardial injury, an ischemic pattern injury (LGE) was identified in 5, dual pathology (ischemic and non-ischemic) in 4, and non-ischemic in 11. Intriguingly, T1 and T2 were not abnormal in this study. This study thus also supports the histological finding of significant myocardial fibrosis but suggests that edema clears fairly quickly in those that recover (2). Again, Rajpal et al. performed CMR on 26 athletes with a history of mild COVID-19 infection. Four of them (15%) had criteria of myocarditis despite mild or no symptoms, and 30% showed signs of previous cardiac injury (65).

### What Can We Conclude From Integrating All Available Data?

Merging the clinical, investigational, and autopsy data, we are presented with a picture that demonstrates a high prevalence of cardiac abnormalities, in part due to exacerbation of underlying cardiac pathology and partly coagulation disorders affecting the pulmonary and coronary vessels. Direct cardiac involvement mainly takes the form of non-coronary myocyte death, myocyte dysfunction, and interstitial fibrosis without substantial inflammatory infiltration or clear ischemia.

The role of direct viral cellular damage remains to be fully explored, and if this is the driving force, it is intriguing that the inflammatory response appears muted. However, it is possible that while the virus is rarely causing a fulminant or acute myocarditis, it can cause a persistent chronic myocardial inflammation with significant long-term implications. It is also important to note the reporting of a delayed immune response in the form of Kawasaki's disease in pediatric patients supporting the issue of long-term sequelae of the SARS-CoV-2 infection (66). Whether immunosuppressive treatment (e.g., dexamethasone and Tocilizumab) during the acute illness is of benefit or causes more harm to the heart should await randomized controlled studies including long-term follow-up.

Thus, on balance, the data strongly suggest significant viral replication in the myocardium without true acute myocarditis in most instances, with frequent non-MI pattern fibrosis—consistent with microvascular ischemia/thrombi and, in some cases, endothelial inflammation. Given the frequent presence of fibrosis associated with cell death, it is likely that complete recovery is unlikely—a clear distinction from septic cardiomyopathy. In addition, the exacerbation of underlying disease would appear to frequently unmask coronary disease, further increasing the benefit of careful cardiological follow-up.

As the vast majority of studied patients in this review died during the acute illness and cardiac abnormality was prevalent in the population studied, we can conclude that myocarditis was not a dominant cause of cardiac dysfunction identified premortem in COVID-19 patients, while the role of endothelialitis needs further clarification.

### Limitations

Our work delineates the importance of PM to guide the understanding of COVID-19. However, the small number of published PM cases in a disease, which has caused more than 1 million fatalities, highlights a hugely missed opportunity. Cardiac pathological changes are more likely to be focal in nature and hence easily missed if the heart is not examined in its entirety. Furthermore, the high prevalence of myocardial fibrosis, myocyte damage, or viral RNA in some studies but not others suggest a need to standardize histological reporting to establish common ground between pathologists and clinicians. There is also a genuine need for an international case register to gather the largest possible data in the shortest interval.

While our work is limited by the quality and small number of cases per study, we think it can contribute to a better understanding of COVID-19-associated cardiac injury. Other limits include the probable selection and reporting bias. PM is performed for patients who died during the acute illness and for certain subgroups of patients due to clinical or legal reasons. The longest duration of illness in our cohort is 52 days, which means that the long-term evolution or complications of the disease cannot be covered by this review.

## Conclusions

To conclude, our review confirmed the high prevalence of cardiac pathological findings in COVID-19 patients. Cardiac dilatation, ischemia, and thrombosis were the most prevalent findings. SARS-CoV-2 was present in nearly half of the examined hearts, but true myocarditis was evident in just 1.5% of the deceased patients.

## Data Availability Statement

The original contributions presented in the study are included in the article/Supplementary Material, further inquiries can be directed to the corresponding author/s.

## Author Contributions

AR: conceptualization and design, registration of the protocol, conduct of the search, quality assessment, data extraction, data interpretation, and manuscript drafting. SZ: conceptualization, design, and writing of the protocol, extraction and interpretation of the data, and manuscript drafting. HF: data analysis and interpretation and writing and revising the manuscript. JC: data analysis and interpretation and writing and reviewing the manuscript. All authors: contributed to the article and approved the submitted version.

## Conflict of Interest

JC received grants and personal fees from Actelion, GSK, Bayer, Endotronix, Pfizer, and United Therapeutics. AR has minor shares in Gilead Sciences. The remaining authors declare that the research was conducted in the absence of any commercial or financial relationships that could be construed as a potential conflict of interest.
