# Supplementary material for: COVID-19 and the Heart: A Systematic Review of Cardiac Autopsies
Source: Front Cardiovasc Med. 2021 Jan 28;7:626975. doi: 10.3389/fcvm.2020.626975 (PMC7876291; doi:10.3389/fcvm.2020.626975)
Supplement: Supplementary file 1 [file Table_1.DOCX]

| **Study** | **Type of study** | **Country** | **n** | **Co-morbidities** | **Days from symptoms to admission + admission to death** | **Clinical Course** | **Cause of death** | **Notes** |
| --- | --- | --- | --- | --- | --- | --- | --- | --- |
| **Duarte-Neto et al ^(6)^** | Case series | Brazil | 10 | M: F = 5:5  **Age**: median 69 years (range: 33-83 years)  **Co-morbidities:**  HTN 5  DM 5  CKD 1  COPD 3  Malignant disease: 1  Chronic cardiopathy 5 | N/A | N/A | N/A | Among 10 cases, one COVID-19 diagnosis based on radiological and pathological findings |
| **Schaller et al ^(12)^** | Case series | Germany | 10 | P1: CML, hypothyroidism  P2: Arteriosclerosis, HTN, AF, CLL, CAD  P3: HTN, arteriosclerosis, COPD, DM, fatty liver disease  P4: HTN, AF, CKD, DC, hypothyroidism, morbid  obesity  P5: Hypertrophic cardiomyopathy  P6: HTN, arteriosclerosis, AF  P7: Adenocarcinoma of the lung  (stage IV), HTN, CKD, hyperthyroidism  P8: COPD, CKD, DM, morbid obesity  P9: HTN, arteriosclerosis, AF, dementia  P10: HTN, arteriosclerosis  7 Males+3 Females | P1: 7+26  P2: 14:15  P3: 7+7  P4: 21+9  P5: 4+8  P6:6+3  P7:5+7  P8:2+5  P9: 3+1  P10: 2+8 | P1: IMV  P2: IMV  P3: IMV  P4: IMV  P5: no IMV  P6: no IMV  P7: no IMV  P8: no IMV  P9: no IMV  P10: no IMV | N/A | 4 mechanically ventilated |
| **Buja et al ^(13)^** | Case series | USA | 3 | P1: 62 M, obese (BMI 33.8)  P2: 34 M, obese (BMI 51.65), HTN, DM2, CHF (LVEF<20%), microcytic anemia.  P3: 48 M, obese (BMI 35.2) | P1: few days +0  P2: 4+10  P3: n/a +0 | P1: OOHCA and death  P2: Oxygen therapy, multiple PE  P3: OOHCA and death | N/A | (discussed 23 including Barton et al 2 cases) |
| **Yan et al ^(14)^** | Case report | USA | 1 | 44 F  Obese (BMI 45.1), ? SLE | 7+6 | Hypoxia, IMV, ICU | N/A | Echo: severe septal, mid anterolateral and mid-inferior hypokinesis; apical and infero-lateral wall motion was preserved. Mildly to  moderately depressed LV systolic function with an estimated LVEF 40-45%. LV size and wall thickness were normal. The patient was diagnosed with reverse Takotsubo cardiomyopathy with clinical suspicion of viral myocarditis. |
| **Lax et al ^(10)^** | Prospective study (Case series) | Austria | 11 | P1: M, HTN, DM, CAD, CVA  P2: M, HTN, DM, CVA  P3: F, HTN, CVA  P4: M, HTN, CAD, malignant disease  P5: M, malignant disease  P6: M, HTN, CAD, CVA, COPD  P7: F, HTN, DM, COPD, dementia  P8: F, HTN, DM, dementia  P9:M, dementia  P10: M, HTN, DM, dementia  P11:M, HTN  **Co-morbidities**  9/11 hypertension  5/11 Diabetes  3/11 coronary artery disease  2/11 previous malignant disease  2/11 COPD  4/11 CVD  4/11 Dementia  Age ranged 75-91 years, (median, 80.5 years; mean, 81.5 years)  Two patients were considered obese,  of whom 1 had a BMI of 33 kg/m2; the other 9 were  considered in good nutritional status, 2 of whom had a  BMI of 27 kg/m2 and 30 kg/m2. | P1:0+4  P2:0+9  P3:0+6  P4:4+7  P5:5+6  P6:2+8  P7:0+4  P8:1+5  P9:0+6  P10:1+9  P11:7+11 | N/A | N/A |  |
| **Lacy et al ^(15)^** | Case report | USA | 1 | 58 F  DM2, obesity (BMI=38), hyperlipidemia, mild intermittent asthma, and chronic LL swelling with ulceration. | 7+0 | OOHCA and death | **After autopsy:**  ARDS due to viral pneumonia due to COVID-19 |  |
| **Wichmann et al ^(16)^** | Prospective cohort study. | Germany | 12 | See table ***  all cases except for case 6 presented  with pre-existing heart disease, including high grade coronary artery sclerosis (7 of 12); myocardial scarring, indicating ischemic heart disease (6 of 12); and congestive cardiomyopathy.  **P1:** 52 M, BMI 38.8, Obesity  **P2:** 70 M, BMI 22.2, PD, CAD, PAD, CKD  **P3:** 71 M, BMI 36.8, HTN, nicotine abuse, granulomatous pneumopathy  **P4:** 63 M, BMI 37.3, DM2, Obesity, bronchial asthma  **P5:** 66 M, BMI 25.3, CAD  **P6:** 54F, BMI 29.6, Trisomy 21, dementia, epilepsy  **P7:**75 F, BMI 26.3, AF, CAD, nicotine abuse  **P8:** 82 M, BMI 27.8, CAD, PD, DM2  **P9:** 87 F, BMI 15.4, COPD, lung cancer, CAD, CKD  **P10:** 84 M, BMI 20.7, HTN, DM2, UC  **P11:** 85 M, BMI 30, CAD, HTN, bronchial asthma, AF  **P12:** 76 M, BMI 34.4, Obesity | N/A | **P1:** CPR  **P2:** supportive care  **P3:** IMV, shock  **P4:** lysis of RV thrombus, IMV, shock  **P5:** CPR  **P6**: supportive care  **P7:** NIV  **P8:** supportive care  **P9:** supportive care  **P10:** supportive care  **P11:** IMV, shock, RRT  **P12:** IMV, shock, CPR | **Clinical:**  **P1:** SCD  **P2:** RF, pneumonia  **P3:** RF, pneumonia  **P4:** Cardiorespiratory failure, PE  **P5:** SCD  **P6:** RF, aspiration pneumonia  **P7:** RF, viral pneumonia  **P8:** RF, viral pneumonia  **P9:** RF, viral pneumonia  **P10:** RF, viral pneumonia  **P11:** cardiac arrest due to RF  **P12:** PE |  |
| **Menter et al ^(17)^** | Case series | Switzerland | 21 | **P1:** 68 F, HTN, atherosclerosis, obesity (BMI 35), MS  **P2:** 86 M, (BMI 26), HTN, dyslipidaemia, sleep apnea, DM2, tMDS, APML and prostate cancer  **P3:** 96 M, (BMI 23), HTN, CAD, MI, PD, dementia  **P4:**78 M, (BMI 44), HTN, dyslipidaemia, obesity, aortic valve reconstruction, AF, CAD, sleep apnea, smoker, DM2,  **P5**:66 M, (BMI 29), HTN, smoker  **P6:**74 M, (BMI 27), HTN, CAD, DM2, Smoker, COPD, wedge resection of lung, Metastatic prostate carcinoma, CKD, genetic thrombophilia  **P7:**81 F, (BMI 26), CAD, PAD, HTN, smoker.  **P8:**71 M, (BMI 25), PAD, infrarenal aortic aneurysm, CAD, valvulopathy, double bypass  **P9:**88 M, (BMI 28), Heart failure, CAD and hypertensive heart disease, AF, Waldenström`s macroglobulinemia, SCC and basalioma  **P10:**85 M, (BMI 29), Hypertensive cardiomyopathy, eccentric hypertrophy with hyperdynamic EF, AF, Ex-smoker (40py)  **P11:**58 M, (BMI 47), HTN, obesity, atherosclerosis  **P12:**75 M, (BMI 27), Dyslipidaemia, HTN, CAD, DM2, sleep apnea  **P13:**53 M, (BMI 59), HTN, obesity, DM2, Sleep apnea, pneumonia-associated ARDS, causing respiratory failure type 1. Patient on long-term oxygen therapy, Liver cirrhosis, acquired immunosuppression  **P14:**94 F, (BMI 19), HTN, tachycardia-bradycardia syndrome, AF, valvulopathy, dementia  **P15:**89 M, (BMI 26), CAD, HTN, valvulopathy, third-degree atrioventricular block, dyslipidaemia, DM2, Ex-smoker  **P16:**61 F, (BMI 41), HTN, obesity, DM2  **P17:**72 M, (BMI 25), HTN, gout, Sleep apnea, ex-smoker  **P18:**79 M, (BMI 27), HTN, dyslipidaemia, CAD, Alzheimer’s, PD  **P19:** 65 M, (BMI 26), HTN, CAD, STEMI  **P20:**71 M, (BMI 36), HTN, obesity, dyslipidaemia, COPD, gout  **P21:**96 M, (BMI 25), HTN, CAD, dementia | **P1:** n/a+9  **P2:** n/a+5  **P3:** n/a+3  **P4:** n/a+3  **P5:** n/a+9  **P6:** n/a+3  **P7:** n/a+4  **P8:** n/a+0  **P9:** n/a+2  **P10:** n/a+5  **P11:** n/a+7  **P12:** n/a+3  **P13:** n/a+8  **P14:** n/a+0  **P15:** n/a+5  **P16:** n/a+9  **P17:** n/a+12  **P18:** n/a+16  **P19:** n/a+7  **P20:** n/a+4  **P21:** n/a+13 | N/A | **P1:** SARS-CoV-2 associated RF with superimposed bacterial bronchopneumonia  **P2:** SARS-CoV-2 associated RF  **P3:** SARS-CoV-2 associated RF with superimposed bacterial bronchopneumonia  **P4:** SARS-CoV-2 associated cardiorespiratory failure  **P5:** SARS-CoV-2 associated respiratory failure with superimposed bacterial bronchopneumonia leading to multi organ failure  **P6:** SARS-CoV-2 associated RF with superimposed bacterial bronchopneumonia  **P7:** SARS-CoV-2 associated respiratory failure with superimposed bacterial bronchopneumonia  **P8:** SARS-CoV-2 associated respiratory failure with superimposed bacterial bronchopneumonia  **P9:** SARS-CoV-2 associated RF  **P10:** SARS-CoV-2 associated RF leading to multi organ failure  **P11:** SARS-CoV-2 associated RF leading to multi organ failure  **P12:** SARS-CoV-2 associated RF with superimposed bacterial bronchopneumonia  **P13:** SARS-CoV-2 associated RF  **P14:** SARS-CoV-2 associated RF  **P15:** SARS-CoV-2 associated RF  **P16:** SARS-CoV-2 associated RF  **P17:** SARS-CoV-2 associated RF  **P18:** SARS-CoV-2 associated RF  **P19:** SARS-CoV-2 associated RF  **P20:** SARS-CoV-2 associated RF with superimposed bacterial bronchopneumonia  **P21:** SARS-CoV-2 associated RF | PE: 4 cases  Vasculitis 1 case |
| **Varga et al ^(7)^** | Case series | N/A | 3  (2 autopsies – 1 still alive) | **P1:** 71 M, Renal transplant, HTN, CAD  **P2:** 58F, DM, HTN, Obesity | **P1 :** n/a +8  **P2 :** 3+16 | All patients developed intestinal ischemia  P1: IMV, shock, RRT in ICU then palliated  P2: ICU, IMV, RRT, RF. On D16 new inferior ST-segment elevation and  echocardiography demonstrated new inferior akinesia suggestive for an acute right  coronary artery occlusion. | N/A | **P1:** Echo: preserved LVEF, dilated LA (59 ml/m2)  **P2:** Echo: normal sized LV with concentric remodeling and normal LVEF (EF 65%) without RWMA. |
| **Tian et al ^(18)^** | Case series | China | 4 | **P1:** 78 F, CLL  **P4:** 59 M, post renal transplantation (3 months) | P1:22  P2:15  P3:23  P4:52  (reported as duration of clinical course from onset of COVID-19 to death) | All died in hospital | N/A | Only heart samples from 2 patients (P1&4) |
| **Barton et al ^(19)^** | Case series | USA | 2 | **P1:** 77 (M) hypertension, splenectomy, Obesity (BMI 31.8), remote DVT, pancreatitis due to cholelithiasis, OA, TKR, positive ANA serology.  **P2:** 42 (M) Obesity (BMI 31.3), myotonic dystrophy | **P1:** 6+0  **P2:** 2+0 | **P1:** arrest on way to hospital  **P2:** arrest few hours after admission | **Autopsy:**  **P1:** COVID-19, with CAD listed under other contributing factors.  **P2:** Complications of hepatic cirrhosis,  with muscular dystrophy, aspiration pneumonia, and  COVID-19 listed as other significant conditions. |  |
| **Conde et al ^(20)^** | Case report | Spain | 1 | 69 M. Bladder cancer | n/a +3 | NIV then admitted to ICU (AKI and shock) | severe bilateral CAP |  |
| **Edler et al ^(21)^**  **Lindner et al ^(22)^** | Cohort | Germany | 80  (74 pre-mortem and 6 post-mortem) | BMI: average 25.9 kg/m^2^.  38% were overweight or obese (overweight  13 cases, obesity grade 1 six cases, grade 2 five cases, grade 3 six cases).  Range: 52-96 years (average  79.2 years, median 82.4 years).  34 F and 46 M  HTN:25, CAD 45, Cardiac Insufficiency 31, cardiomyopathy 9, AF 15, VHD 3, DM 17, CKD 25, Malignant d 13, COPD 42, OSA 2, CVD 10, Dementia 19, 1 LBBB, 1 A Flutter, 1 Cardiac arrythmia, 1 nephrectomy, 1 renal transplant | N/A | Place of death:  12 home  1: hotel  13: nursing or retirement home.  51: Hospital (17 in ICU+IMV),  31 in ward,  1 in ER). | Pneumonia: 66  Bronchitis and bronchopneumonia: 5  PE: 6  Sepsis: 7  MI: 1  MI+Tamponade: 1  SCD: 2  Cardiac decomp: 1  AV endocarditis: 1  Metastatic lung cancer: 1  Necrotising fasciitis: 1 | 17 PE including 8 fatal; In each of these deaths as well as in 15 others (in total 32 cases, 40%), had LL DVT |
| **Sekulic et al ^(23)^** | Case series | N/A | 2 | **P1:** 81 M, dementia, left lung mass  (managed with hospice care), CAD  (post CABG), AF (biventricular PPM), CHF, PAD (post iliac stenting), DM, HTN, CKD, CVA, UTI, dyslipidemia, gout, carotid endarterectomy, left inguinal hernia repair and cataract surgery.  **P2:** 54 M, HTN, DM2, non-smoker, BMI 29.9 | P1: 8+5  (autopsy 29 hours after death)  P2: 2+10  (autopsy 39 hours after death) | P1: Oxygen therapy then palliative care.  P2: RF, ICU, IMV, D10, PEA same day | **P1:** RF due to SARS-CoV-2  **P2:** SARS-CoV-2 infection leading to  respiratory and multiorgan system failure |  |
| **Suess et al ^(24)^** | Case report | N/A | 1 | 59 M, HTN and DM2, BMI 25.91 | 5+0 | OOHCA and death after 5 days: fever, dry cough and tachycardia | ARDS due to severe DAD as a result of severe infection with SARS CoV-2. |  |
| **Aguiar et al ^(25)^** | Case report | Switzerland | 1 | 31 F, Morbid obesity (BMI 61.2) | 7+0 | OOHCA and death after presenting with cough for 7 days | **Pathology:**  Pulmonary changes related to SARS-CoV-2 and high fever without secondary bacterial infection |  |
| **Fox et al ^(26)^** | Case series | USA | 10 (Heart examination in 9/10) | P1: 44, HTN, DN2, CKD, BMI 37.5  P2: 44, HTN, DM2, CHF, BMI 47.76  P3: 63, HTN, Thyroidectomy, BMI 56.97  P4: 76, HTN, DM2, RA, BMI 38.15  P5: 68, DM2, BMI 35.75  P6: 78, N/A, BMI 33.1  P7:53, HTN, polymyositis, OSA, BMI 28.5  P8: 60, HTN, COPD, AF, BMI 44.8  P9:66, HTN, BMI 28.3  P10: 78, ESRD, DM2, BMI 34.3 | P1: 3+8  P2: 6+5  P3: 7+25  P4: 3+6  P5: 1+9  P6: 7+0  P7: 0+1  P8: 3+14  P9: 2+12  P10: 2+2 | P1: IMV  P2: IMV  P3: IMV  P4: IMV  P5: IMV  P6: hypoxic respiratory failure and VT and cardiac arrest in emergency department  P7: IMV  P8: IMV  P9: IMV  P10: IMV | Cause of death: COVID-19  (Withdrawal of care) | Heart examination in 9 |
| **Beigmohammadi et al ^(27)^** | Case Series | Iran | 7 (5 with cardiac tissues) | P1: 58 M, HTN  P3: 72 F, RA  P5: 68 M. HTN, VHD: Heart valve replacement procedure due to endocarditis about 1 month before his death.  P6: 46 M, PUD  P7: 75 M | P1: n/a +7  P3: n/a +15  P5: 0+11 (already in-hospital)  P6: n/a +16  P7: n/a +6 | All intubated and ventilated | N/A | Author’s conclusion: no myocarditis ischemia is most probable |
| **Wang C et al ^(28)^** | Case Series | China | 2 | **P1:** 53 F, DM and HTN  **P2:**62 M | P1: 20+8  P2: 13+10 | P1: NIV and AKI  P2: O2 therapy | Respiratory and circulatory failure (Both) | 6- and 9-hours death to autopsy |
| **Rapkiewicz et al ^(8)^** | Case series | USA | 7 (4 females) vs 9 controls died from ARDS from other cause | **P1:**64F  HTN, DM, high Cholesterol, obesity (BMI 36), COPD, lung cancer  **P2:**60M  HTN, DM, Obesity (BMI 32.3), CAD  **P3:**50F  HTN, high Cholesterol, DM, BMI (23.0)  **P4:**44M  HTN, high Cholesterol, DM, Obesity (BMI 38.8), RCC  **P5:**64F  HTN, high Cholesterol, Obesity (BMI 35.6), OSA  **P6:**55F, Obesity (BMI 30.7)  **P7:**65M  HTN, DM, (BMI 25.0), Cirrhosis, PAD, CKD, hypothyroidism | P1:3+0  P2:7+0  P3:7+2  P4:7+6  P5:7+9  P6:14+11  P7:14+3 | P1: OOHCA  P2: OOHCA  Died in the community  P3-7: hospitalised, IMV  P7: Large rectal bleed | N/A |  |
| **Bösmüller et al ^(29)^** | Case series | Germany | 4 | **P1:** 78 F, obese (BMI 35.2), HTN, AV block (PPM)  **P2:** 79 M, CAD, HTN, DM2, and PD (BMI 28.4 kg/m^2^).  **P3:** 72 M, CAD, Merkel cell carcinoma under adjuvant radiotherapy, obesity, polymyalgia rheumatica  **P4:** 59 M, HTN, intrinsic asthma | P1: 12 hrs symptoms + 0  P2: 21+9  P3: n/a+16  P4: 14+35 | P1: died at home  P2: general weakness for 3 weeks, fever, and dry cough with worsening symptoms 3 days before admission.  ICU (8 days) – IMV – dialysis  P3: IMV, ICU (11 days) + dialysis  P4: ICU + 24 days ECMO + dialysis | **Clinical**  P1: Pneumonia  P2: ARDS, liver  failure, shock  P3: ARDS, liver  failure, shock  P4: ARDS, multi-organ  failure | Autopsy after 48 hours for patient 1 and within 24 hours for patients 2,3 and 4 |
| **Schweitzer et al ^(30)^** | Case report | Switzerland | 1 (and 1 control) | 50 M  HIV positive  Treated for chest infection 5 weeks earlier | Symptoms to death: 8 days | Out of hospital cardiac arrest and death | N/A  ? severe ARDS |  |
| **Xu et al ^(31)^** | Case report | China | 1 | 50 M | 9+5 | IMV D14 | N/A |  |
| **Youd et al ^(32)^** | Case Series | UK | 3 | **P1:** 88 F, BMI: 29 kg/m2, Dementia  **P2:** 86 M, BMI: 24 kg/m2, HTN, COPD, heart disease, dementia  **P3:** 73 F, BMI: 32 kg/m2, DM1, asthma, heart disease | N/A | All died in community | N/A | (9 cases but only 3 COVID) |
| **Bradley et al ^(9)^** | Case series | USA | 14 | **P1:** 57 M, ESRD, DM, HTN, OSA, obesity  **P2:**74 F, DM, OSA, AF, pulmonary hypertension, CKD, obesity  **P3:**54 M, TBI with secondary neurological  dysfunction and dysphagia  **P4:**74 M, Heart failure with preserved EF,  frontotemporal dementia, HTN, OSA  **P5:**73 F, DM2, HTN, CHF, hypothyroidism, obesity, schizoaffective disorder, bipolar disorder  **P6:**84 F, COPD, CHF, AF, AS, HTN, CKD, osteoporosis  **P7:**71 M, HTN, hyperlipidaemia, CAD, AF, ERSD, OSA  **P8:**76 F, Hyperlipidaemia, osteoporosis  **P9:**75 F, Hyperlipidaemia, DM2, CAD, CHF, CKD, COPD, previous DVT  **P10:**84 M, CKD, COPD, hyperlipidaemia,  OSA, MR, complete heart block, chronic pain, arthritis, obesity, HTN  **P11:**81 F, HTN, hyperlipidaemia, breast cancer, CKD, demyelinating neuropathy, lacunar infarcts, recent pneumonia, Alzheimer’s disease  **P12:**42 F, breast cancer status post-bilateral mastectomy and chemotherapy and anaemia  **P13:**71 M, CAD ischaemic cardiomyopathy,  HTN, AS, ERSD on dialysis, pulmonary fibrosis, previous cerebellar cardiovascular accident  **P14:** 73 F, HTN, asthma, DM, hyperlipidaemia,  obesity | **P1:** 4+6  **P2:** 2+0  **P3:**1+1  **P4:** 1+0  **P5:**5+8  **P6:**1+1  **P7:**7+6  **P8:**3+4  **P9:**3+9  **P10:**1+0  **P11:**1+6  **P12:**5+9  **P13:**1+3  **P14:**2+21 | **P1: IMV**  **P2: IMV**  **P3: n/a**  **P4: IMV**  **P5: IMV**  **P6: n/a**  **P7: n/a**  **P8: IMV**  **P9: n/a**  **P10: n/a**  **P11: IMV**  **P12: IMV**  **P13: n/a**  **P14: IMV** | **P1:** Cause A: COVID-19 pneumonia  **P2:** Cause A: Cardiomyopathy, cause B: COVID-19  **P3:** Cause A: aspiration pneumonia  and sepsis, cause B:  COVID-19 infection  **P4:** Cause A: ARDS, cause B: viral  pneumonia, cause C: COVID-19  **P5:** Cause A: ARDS, cause B: viral  pneumonia, cause C: COVID-19  **P6:** Cause A: ARDS, cause B: viral pneumonia, cause C:  COVID-19  **P7:** Cause A: viral pneumonia, cause B: COVID-19, cause C: immunosuppression **cause D: renal transplant**  **P8:** Cause A: ARDS, cause B: viral  Pneumonia, cause C:  COVID-19  **P9:** Cause A: ARDS, cause B: pneumonia, cause C: COVID-19  **P10:** Cause A: acute or chronic hypoxic  and hypercarbic RF, cause B: pulmonary  emphysema  P11: Cause A: acute hypoxic respiratory  failure, cause B: ARDS, cause C: co-incident viral and bacterial pneumonia, cause D: COVID-19  **P12:** Cause A: ARDS, cause B: COVID-19  **P13:** Cause A: ventricular fibrillation  (I49.01), cause B: ARDS, cause C: COVID-19 respiratory infection  **P14:** Cause A: ARDS, cause B:  COVID-19 pneumonia | Excluding other significant conditions from cause of death |
| **Ducloyer et al ^(33)^** | Case report | France | 1 | 75 (M)  BMI: 25  No past medical history | 9+0 | Complaint: Fever, moderate asthenia, diarrhoea and drowsiness.  Died in community | Severe lung damage |  |
| **Cirstea et al ^(34)^** | Case report | Romania | 1 | 30F  No comorbidities | 13+0 | Died at home | N/A |  |
| **Nicolai et al ^(35)^** | Case control | Germany | 5 cases (and 5 control)  ***But only one case had heart tissue*** | **P1:** 91 years | P1:?+7 | **P1:** ARDS & died in ward (declined ICU) | N/A | Excluded: severe pre-existing kidney or liver dysfunction, severe autoimmune diseases, immunosuppression, chronic infection, patients  requiring ECMO therapy, with a known coinfection with Influenza or Respiratory Syncytial  Virus (RSV) and patients receiving antiplatelet medication. |
| **Grosse et al ^(36)^** | Case series | Austria | 14 | **Co-morbidities:**  **CAD:** 14/14 (previous MI in 2/14)  **Cardiomyopathy:** 5/14  **HTN:** 8/14  **Dementia:** 5  **Stroke:** 2  **CKD:** 7  **DM:** 5  **COPD:** 6  **DVT:** 1  **Malignancy:** 3  **P1:** 81M, heart disease, CKD, DM, malignancy, respiratory disease, liver cirrhosis/fibrosis  **P2:** 71M, heart disease, neurologic disease, respiratory disease, chronic gastritis  **P3:** 75M, heart disease, HTN, CKD, malignancy, respiratory disease  **P4:** 94F, heart disease, HTN, CKD, neurologic disease  **P5:** 55M, heart disease  **P6:** 81M, heart disease, HTN, neurologic disease and chronic gastritis  **P7:** 87M, heart disease, HTN, respiratory disease  **P8:** 83F, heart disease, HTN, neurologic disease, chronic gastritis  **P9:** 90F, heart disease, CKD, neurologic disease, chronic gastritis  **P10:** 84F, heart disease, CKD, DM  **P11:** 80F, heart disease, HTN, CKD, neurologic disease, DM, chronic gastritis  **P12:** 72M, heart disease, HTN, DM, chronic gastritis  **P13:** 94M, heart disease, HTN, neurologic disease, respiratory disease  **P14:** 82M, heart disease, CKD, DM, malignancy, respiratory disease | Symptoms to death reported  **P1: ?+?=6**  **P2: ?+?=7**  **P3: ?+?=10**  **P4: ?+?=12**  **P5: ?+?=16**  **P6: ?+?=17**  **P7:** ?+?=19  **P8:** ?+?= 21  **P9**:?+?=21  **P10:?+?=23**  **P11**:?+?=28  **P12**:?+?=29  **P13**:?+?=30  **P14**:?+?=50 | 7 patients acquired COVID during hospitalisation  7 patients I+V in ICU  Superimposed bronchopneumonia in 11/14 | Acute bronchopneumonia:  2 patients  Acute MI: 3 patients  Fungal sepsis : 1 patient | ***5 types of pulmonary thrombi:***  1. Capillary microthrombi (11/14)  2. Partially organized thrombi in mid-sized PA with complete vessel occlusion;  3. Non-organized thrombi in mid-sized PA – no complete occlusion  4. bone marrow emboli (1/14)  5. septic pulmonary thromboemboli (1/14). |
| **Shwensen et al ^(37)^** | Case report | Denmark | 1 | 80s F  Atherosclerosis  CAD: stable angina  TIA  Breast cancer  Normal Echocardiography | 7+45 | Flu like symptoms for 7 days  ICU  Tracheostomy  Withdrawal | N/A | May represent long term sequelae of COVID-19 |
| **Remmelink et al ^(38)^** | Case series | Belgium | 17 | **P1:** 77M, CAD, CVD, DM  **P2:** 91F, HTN, CAD, CKD, liver cirrhosis  **P3:** 68M, COPD, cancer  **P4:** 64F, HTN, cancer, CVD  **P5:** 56M, COPD, cancer  **P6:** 73M, HTN, CKD  **P7:** 56M, none  **P8:** 66M, CAD, HTN, DM, CKD, CVD  **P9:** 49F, HTN, DM  **P10:** 63M, HTN, DM  **P11:** 76M, DM, cancer, liver cirrhosis  **P12:** 75M, HTN, CAD, DM  **P13:** 73M, DM  **P14:** 77F, HTN, DM  **P15:** 61M  **P16:** 70F, HTN, DM  **P17:** 53M, HTN, CVD  **Total:**  HTN: 10  CAD: 4  CVD: 4  DM: 9  Cancer: 4 | **P1:?+3**  **P2:?+15**  **P3:?+15**  **P4:?+8**  **P5:?+14**  **P6:?+11**  **P7:?+7**  **P8:?+14**  **P9:?+17**  **P10:?+16**  **P11:?+5**  **P12:?+6**  **P13:?+10**  **P14:?+9**  **P15:?+31**  **P16:?+19**  **P17:?+13**  Duration between onset of symptoms and death: range 2 to 40 days (median, 14 days) | 11 died in ICU  6 died on ward  **P1:** I+V, ARDS, AKI  **P2:** ARDS, AKI, Hydroxychloroquine, antibiotics, CS  **P3:** I+V, ARDS, AKI  **P4: I+V, ARDS**  **P5:** I+V, ECMO, RRT, ARDS, AKI  **P6:** I+V, ECMO, ARDS, AKI  **P7:** ARDS, AKI  **P8:** AKI  **P9:** ARDS, AKI, I+V, RRT  **P10:** I+V, ARDS, AKI, ECMO, RRT  **P11:** ARDS  **P12:** I+V, ARDS, AKI  **P13:** ARDS  **P14:** I+V, RRT, ARDS, AKI  **P15:** I+V, RRT, ARDS, AKI, PE  **P16:** I+V, RRT, ARDS, AKI, PE  **P17:** I+V, RRT, ECMO, ARDS, AKI, PE | *As documented by attending physician*  **P1:** Cardiogenic shock, MOF  **P2:** Respiratory failure  **P3:** Respiratory failure  **P4:** Respiratory failure  **P5:** Mesenteric ischemia, MOF  **P6:** Respiratory failure  **P7:** Respiratory failure  **P8:** Septic shock, MOF  **P9:** Respiratory failure  **P10:** Respiratory failure  **P11:** Sudden death  **P12:** MOF  **P13:** Respiratory failure  **P14:** Respiratory failure  **P15:** Septic shock, MOF  **P16:** Septic shock, MOF  **P17:** Septic shock, MOF | Troponin documented |
| **Okudela et al ^(39)^** | Case report | Japan | 1 | 93F  PMH: N/A | 10+10 | N/A | N/A |  |
| **Adachi et al ^(40)^** | Case report | Japan | 1 | 84 F  PMH: none | 8+16 | Progressive respiratory failure, declined mechanical ventilation then palliation in hospital | N/A |  |
| **Nadkarni et al ^(41)^** | Retrospective  (case series) | USA | 26 autopsies (focus on thromboembolism) | M: 16  F: 10  AF: 3  DVT: 1 | **P1:** ?+9  **P2:** ?+11  **P3:** ?+4  **P4:** ?+6  **P5: ?+0**  **P6: ?+7**  **P7: ?+10**  **P8:?+10**  **P9:?+0**  **P10:?+0**  **P11:?+1**  **P12:?+3**  **P13:?+1**  **P14:?+1**  **P15:?+4**  **P16:?+5**  **P17:?+1**  **P18:?+5**  **P19:?+6**  **P20:?+4**  **P21:?+5**  **P22: ?+15**  **P23:?+10**  **P24:?+9**  **P25:?+22**  **P26:?+11** | N/A | N/A | N/A |
| **Dalahmah et al ^(42)^** | Case report | USA | 1 | 73M  HTN, DM2 | 0+1 | OOHCA  I+V | Acute cerebellar haemorrhage |  |
| **Oprinca et al ^(43)^** | Case series | Romania | 3 | **P1:** 79F, HTN, CAD, AF  **P2:**27M, OSA, Obesity, smoker  **P3:**70M, HTN, CAD (aortocoronary bypass), CHF, AF, Mitral and tricuspid regurge, CKD, atherosclerosis, chronic rhinitis and pharyngitis | **P1:**2+?  **P2:**6+0  **P3**:?+1 | **P1:** Pneumonia, AKI  **P2:** died at home  **P3:** GI bleed then cardiac arrest | **P1:** Lung injury due to viral pneumonia  **P2:** Lung injury due to viral pneumonia  **P3:** Intestinal haemorrhage due to anticoagulant drug overdose | N/A |
| **Wang X et al ^(44)^** | Case series | China | 3  (only 1 had biopsy of the heart) | **P3:**75F, CAD, HTN, DM2, hyperlipidaemia | **P3: ?+10** | **P3:** Died in hospital; high troponin | **P3:** MOF | N/A |
| **Jensen et al ^(45)^** | Case series | N/A | 2 | **P1:** 71M  **P2:** 66M, HTN, CAD, hypertensive heart disease, Overweight, COPD, DM | **P1:**14+30  **P2:**?+30 | **P1:** I+V, ICU, AKI, RRT  **P2:** ICU, RRT, I+V | **N/A** | N/A |
| **Elsoukkary et al ^(46)^** | Case series | USA | 32 (but only 30 had heart examination) | Age: Mean 68 years, range 30-100  Males: 22  Females: 10  HTN: 21/30  CAD: 10/30  CHF: 6/30  DM:20/32  Obesity 10/32 | Average duration till time of death: 21 days  Range: 1-58 days | ICU: 17  No ICU: 15  1 died at home  I+V: 15  AKI: 16  RRT: 6 | N/A | N/A  NB: Normal heart weight:  male 270–360 g,  Female 200–280 g.  23/30 had elevated troponin |
| **Hanley et al ^(47)^** | Case series | UK | 10 (including one limited biopsy) | **Male:7**  **Female:3**  **Co-morbidities:**  HTN:4  COPD:3  Obese:5 (Median BMI 31.2, IQR 22.3-40)  **P1:** 61M, CAD, COPD, BMI 33.1  **P2:** 64M, OSA, Migraine, Prostatic hyperplasia and hernia repair, BMI 35.86  **P3:** 69F, COPD, OSA, Corpulmonale, CAD, HTN, DM2, peripheral neuropathy, smoking, Obesity, BMI 44.1  **P4:** 78M, Dementia, HTN, DM2, OA, BMI 24.7  **P5:** 22M, Obesity, hypothyroidism, BMI 48.8  **P6:** 24M, non-alcoholic steatohepatitis, lichen planus and Gonadotrophin releasing hormone deficiency, BMI 25.2  **P7:** 79M, hypercholesterolemia and trigeminal neuralgia, BMI 31.2  **P8:** 97M, UTI, dementia, bladder cancer, anaemia, hypothyroidism, glaucoma, alcohol-related liver disease and PPM, BMI 18.3  **P9:** 79F, COPD, cutaneous systemic lupus  erythematosus, HTN, DM2 and vitamin B12 deficiency, BMI 19.72  P10: 77F, HTN, LVH, osteoporosis, OA, CKD, vasculitis, hypercholesterolemia, hiatus hernia, pancreatitis, b12 and vitamin D deficiency | **P1:7+3**  **P2:?+?=13**  **P3:5+3**  **P4:4+8**  **P5: ?+?=27**  **P6: ?+?=8**  **P7: 7+16**  **P8:?+?=23**  **P9: 7+17=24**  **P10:14+1** | I+V: 4 patients  P1: OOHCA  P2: ICU  P3: ward, palliated  P4: ward, palliated  P5: ICU, MCA infarct and haemorrhage, pericardial effusion drained.  P6: ICU, I+V  P7: ICU. I+V  P8: Ward, palliated  P9: Ward, palliated  P10: ward, palliated | **P1:**  **Clinical:**  1a COVID-19 pneumonia  2 COPD, IHD  **After autopsy:**  1a DAD and MI  1b SARS-CoV-2 infection and coronary artery atherosclerosis (stented)  2 IHD, liver, elevated BMI  **P2:**  **Clinical:**  1a COVID-19 pneumonitis  2 OSA  **After autopsy:**  1a DAD  1b SARS-CoV-2 infection  2 OSA  **P3:**  **Clinical:**  1a COVID-19 Pneumonitis  2 COPD, OSA, IHD, HTN, obesity, smoker, DM2  **After autopsy:**  1a Pulmonary oedema and DAD  1b SARS-CoV-2 infection  2 Obesity, HTN, DM2, smoker, IHD, OSA  **P4:**  **Clinical:**  1a COVID-19 Pneumonia  2 Dementia, frailty, HTN  **After autopsy:**  1a DAD and haemophagocytosis  1b SARS-CoV-2 infection  2 Dementia, frailty, HTN.  **P5:**  **Clinical:**  1a COVID-19 pneumonia  2 Cerebral infarct, hypothyroidism  **After autopsy:**  1a MOF  1b Disseminated Mucormycosis  1c SARS-CoV-2 Infection  2 Elevated BMI, hypothyroidism, steatohepatitis  **P6:**  **Clinical:**  1a MOF  1b Pneumonia – COVID-19  2 Non-alcoholic steatohepatitis  **After autopsy:**  1a DAD  1b SARS-CoV-2 infection  **P7:**  **Clinical:**  1a SARS-COVID-19  **After autopsy:**  1a DAD  1b SARS-CoV-2 Infection  2 HTN, CKD, LVH, coronary atherosclerosis.  **P8 :**  **Clinical:**  1a Pneumonia – COVID-19  2 Advanced frailty, dementia, liver cirrhosis, bladder cancer (treated).  **After autopsy:**  1a DAD and widespread thrombosis  1b SARS-CoV-2 Infection  2 Early hepatic fibrosis, dementia, frailty and cardiac amyloidosis  **P9:**  **Clinical:**  1a Pneumonia (COVID-19)  2 HTN, DM2, COPD  **After autopsy:**  1a DAD  1b SARS-CoV-2 Infection  2 HTN, DM2, COPD.  **P10:**  **Clinical:**  1a SARS-COVID-19  1a COVID-19 Pneumonia  2 ANCA-associated vasculitis, pulmonary fibrosis, HTN, CKD  **After autopsy:**  1a DAD  1b SARS-CoV-2 Infection  2 HTN, CKD, LVH, coronary atherosclerosis. | All full biopsy except P10 |

Ab: antibodies, AF: Atrial fibrillation, ANA: antinuclear antibody; ARDS: acute respiratory distress syndrome; AS: aortic stenosis; AV: Atrioventricular; BMI: Body Mass index; CABG: coronary artery bypass grafting; CA: coronary artery; CAD: Coronary artery disease; CAP: Community acquired pneumonia; CHF: Congestive heart failure, CKD: Chronic Kidney disease, CLL: Chronic Lymphocytic leukaemia; CMC: cardiomyocytes, CML: Chronic myelomonocytic leukaemia ; COPD: Chronic Obstructive pulmonary disease; CS: Corticosteroids; CVA: cerebrovascular accidents; DAD: diffuse alveolar damage; DC: dilated cardiomyopathy DM: Diabetes Meletus; DVT: Deep venous thrombosis; ECG: Electrocardiogram, EM: Electron Microscopy; ER: Emergency room; ESRD: End-stage renal disease, HTN: Hypertension; ICU: Intensive care unit; IHC: Immunohistochemistry; IHD: Ischemic heart disease; IMV: Invasive mechanical ventilation; IVS: Interventricular septum; LV: Left ventricle; LVH: left ventricular hypertrophy; LVEF: Left ventricular ejection fraction; MI: Myocardial infarction; MOF: Multi-organ failure; MR: Mitral regurgitation; MS: Multiple sclerosis; N/A: Not available; OA: Osteoarthritis, OSA: Obstructive sleep apnea, PA: Pulmonary artery; PAD: Peripheral arterial disease, PE: Pulmonary embolism; PD: Parkinson disease; PPM Permanent Pacemaker; PUD: Peptic Ulcer disease, RF: respiratory failure; RRT: renal replacement therapy; RWMA: regional wall motion abnormalities; SCD: Sudden cardiac death; SLE: systemic lupus erythematous; TBI: Traumatic brain injury; TKR: Total knee replacement; RA: Rheumatoid arthritis, RCC: Renal cell carcinoma; RF: respiratory failure; RRT: Renal replacement therapy; RV: Right ventricle; UC: Ulcerative colitis; UK: United Kingdom; USA: United states of America; UTI: urinary tract infections; VHD: Valvular heart disease
