# Supplementary material for: COVID-19 and the Heart: A Systematic Review of Cardiac Autopsies
Source: Front Cardiovasc Med. 2021 Jan 28;7:626975. doi: 10.3389/fcvm.2020.626975 (PMC7876291; doi:10.3389/fcvm.2020.626975)
Supplement: Supplementary file 2 [file Table_2.DOCX]

**Supplementary table 2: Quality assessment by the JBI tool for the case series reporting cardiac autopsy studies**

| **Study** | Were there clear criteria for inclusion in the case series? | Was the condition measured in a standard, reliable way for all participants included in the case series? | Were valid methods used for identification of the condition for all participants included in the case series? | Did the case series have consecutive inclusion of participants? | Did the case series have complete inclusion of participants? | Was there clear reporting of the demographics of the participants in the study? | Was there clear reporting of clinical information of the participants? | Were the outcomes or follow up results of cases clearly reported? | Was there clear reporting of the presenting site(s)/clinic(s) demographic information? | Was statistical analysis appropriate? | Include/  exclude/  further |
| --- | --- | --- | --- | --- | --- | --- | --- | --- | --- | --- | --- |
| Duarte-Neto et al ^(6)^ | No* | No | No | Yes | Yes | Yes | Yes | Yes | Yes | N/A | Include |
| Schaller et al ^(12)^ | Yes | Yes | Yes | Yes | No | No | No | Yes | Yes | N/A | Include |
| Buja et al ^(13)^ | Unclear | Unclear | Unclear | No | No | Yes | No | Yes | Yes | N/A | Include |
| Lax et al ^(10)^ | Yes | Yes | Yes | No | No | Yes | Yes | Yes | Yes | N/A | Include |
| Wichmann et al ^(16)^ | Yes | Yes | Yes | Yes | Yes | Yes | Yes | Yes | Yes | N/A | Include |
| Menter et al ^(17)^ | Yes | Yes | Yes | Unclear | Unclear | Yes | Yes | Yes | Yes | N/A | Include |
| Varga et al ^(7)^ | Yes | Unclear | Yes | Unclear | No | Yes | Yes | Yes | No | N/A | Include |
| Tian et al ^(18)^ | Yes | Yes | No | Unclear | Unclear | Yes | Yes | Yes | Yes | N/A | Include |
| Barton et al ^(19)^ | Yes | Yes | Yes | Unclear | Unclear | Yes | Yes | Yes | Yes | N/A | Include |
| Edler et al ^(21)^ | Yes | Yes | Yes | No | Yes | Yes | No | Yes | Yes | N/A | Include |
| Sekulic et al ^(23)^ | Yes | Yes | Yes | Unclear | No | Yes | Yes | Yes | No | N/A | Include |
| Fox et al ^(26)^ | Yes | Yes | Yes | Unclear | Unclear | Yes | Yes | Yes | Yes | N/A | Include |
| Beigmohammadi et al ^(27)^ | Yes | Yes | Unclear | Unclear | Yes | Yes | Yes | Yes | Yes | N/A | Include |
| Wang C et al ^(28)^ | Yes | Yes | Yes | Unclear | No | Yes | Yes | Yes | Yes | N/A | Include |
| Rapkiewicz et al ^(8)^ | Yes | Yes | Yes | Yes | Unclear | Yes | Yes | Yes | Yes | N/A | Include |
| Bösmüller et al ^(29)^ | Yes | Yes | Yes | No | No | Yes | Yes | Yes | Yes | N/A | Include |
| Youd et al ^(32)^ | Yes | Yes | Yes | Unclear | Unclear | Yes | Yes | Yes | Yes | N/A | Include |
| Bradley et al ^(9)^ | Yes | Yes | Yes | Unclear | Unclear | Yes | No | Yes | Yes | N/A | Include |
| Grosse et al ^(36)^ | Yes | Yes | Yes | Yes | No | Yes | Yes | Yes | Yes | N/A | Include |
| Remmelink et al ^(38)^ | Yes | Yes | Yes | Yes | No | Yes | Yes | Yes | Yes | N/A | Include |
| Nadkarni et al ^(41)^ | Yes | Yes | Unclear | Yes | No | No | No | No | Yes | N/A | Include |
| Oprinca et al ^(43)^ | Yes | Yes | Yes | No | No | Yes | Yes | Yes | No | N/A | Include |
| Wang X et al ^(44)^ | Yes | No | No | No | No | Yes | Yes | Yes | No | N/A | Include |
| Jensen et al ^(45)^ | Yes | No | Yes | No | No | Yes | Yes | No | No | N/A | Include |
| Elsoukkary et al ^(46)^ | Yes | Unclear | Yes | No | No | No | No | Yes | No | N/A | Include |
| Hanley et al ^(47)^ | Yes | No | Yes | No | No | Yes | Yes | Yes | Yes | N/A | Include |

N/A not applicable

*One case was included based on radiological and pathological findings
